# Supplementary material for: Assessing the co-variability of DNA methylation across peripheral cells and tissues: Implications for the interpretation of findings in epigenetic epidemiology
Source: PLoS Genet. 2021 Mar 19;17(3):e1009443. doi: 10.1371/journal.pgen.1009443 (PMC8011804; doi:10.1371/journal.pgen.1009443)

Figure S1. Heatmap of the first ten DNA methylation principal components across the five purified blood cell types and three peripheral tissue samples (whole blood, buccal epithelial cells and nasal epithelial cells) profiled in this study. Shown is the mean principal component value for samples grouped by cell- or tissue-type. Each row represents a principal component (PC1 to PC10), with the percentage of variance in DNA methylation by each explained in brackets. Each column represents a single sample type.

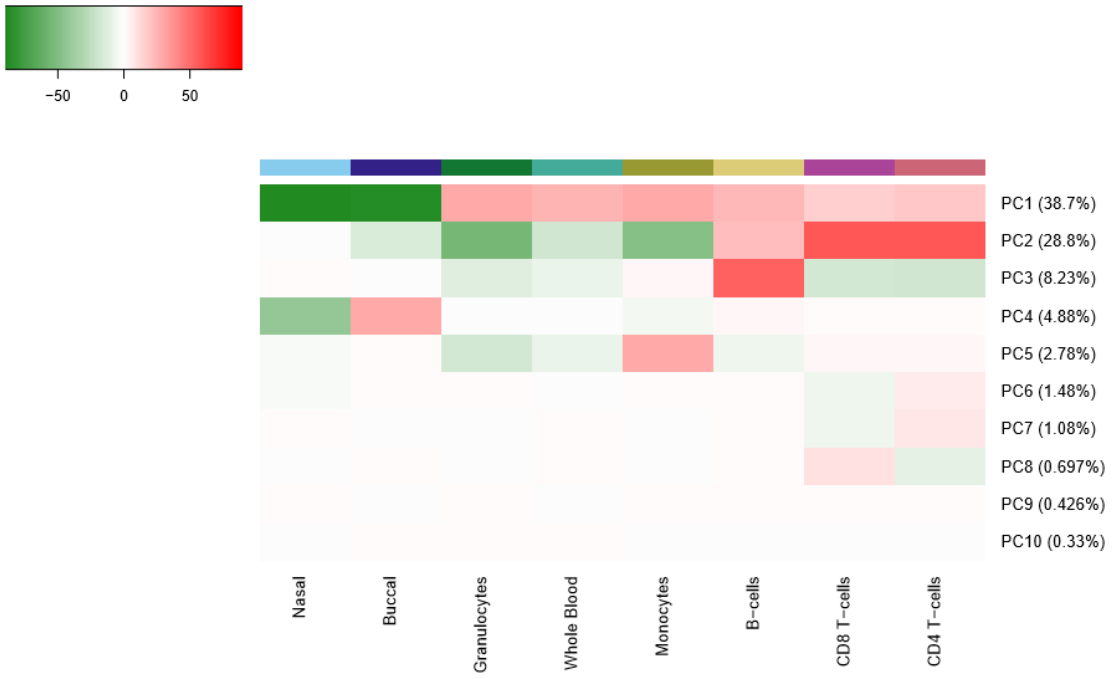

Supplement: S1 Fig — Shown is the mean principal component value for samples grouped by cell- or tissue-type. Each row represents a principal component (PC1 to PC10), with the percentage of variance in DNA methylation by each explained in brackets. Each column represents a single sample type. (PDF) [file pgen.1009443.s001.pdf]
