# Supplementary material for: Assessing the co-variability of DNA methylation across peripheral cells and tissues: Implications for the interpretation of findings in epigenetic epidemiology
Source: PLoS Genet. 2021 Mar 19;17(3):e1009443. doi: 10.1371/journal.pgen.1009443 (PMC8011804; doi:10.1371/journal.pgen.1009443)

Figure S2. Density plot of DNA methylation levels across the 784,726 autosomal DNAm sites included in our analysis for each sample type. Shown is the mean level of DNAm at each site across all individuals.

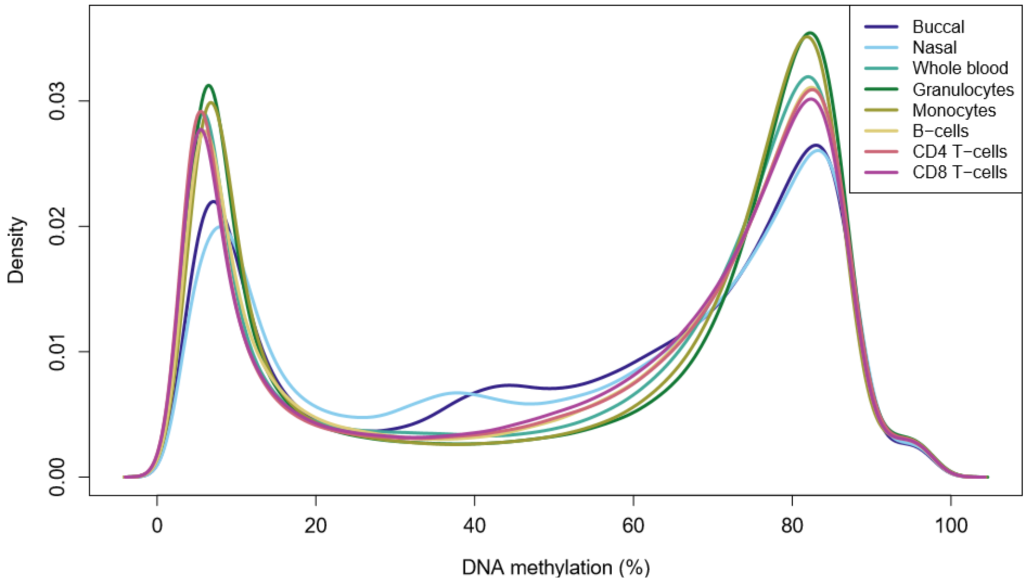

Supplement: S2 Fig — Shown is the mean level of DNAm at each site across all individuals. (PDF) [file pgen.1009443.s002.pdf]
