# Supplementary material for: Assessing the co-variability of DNA methylation across peripheral cells and tissues: Implications for the interpretation of findings in epigenetic epidemiology
Source: PLoS Genet. 2021 Mar 19;17(3):e1009443. doi: 10.1371/journal.pgen.1009443 (PMC8011804; doi:10.1371/journal.pgen.1009443)

Figure S3. Bar chart showing the proportion of differentially methylated positions (DMPs) compared to whole blood shared between different sample types. For each sample type the sites identified as differentially methylated relative to whole blood were categorized into those that are uniquely different in that sample type or shared with at least one other sample type. Unique DMPs were defined as those where the t-statistic comparing each sample type to whole blood were significant for only a single sample-type. Bar chart **A)** shows the number and **B)** shows the percentage of unique and shared DMPs compared to whole blood for each sample type.

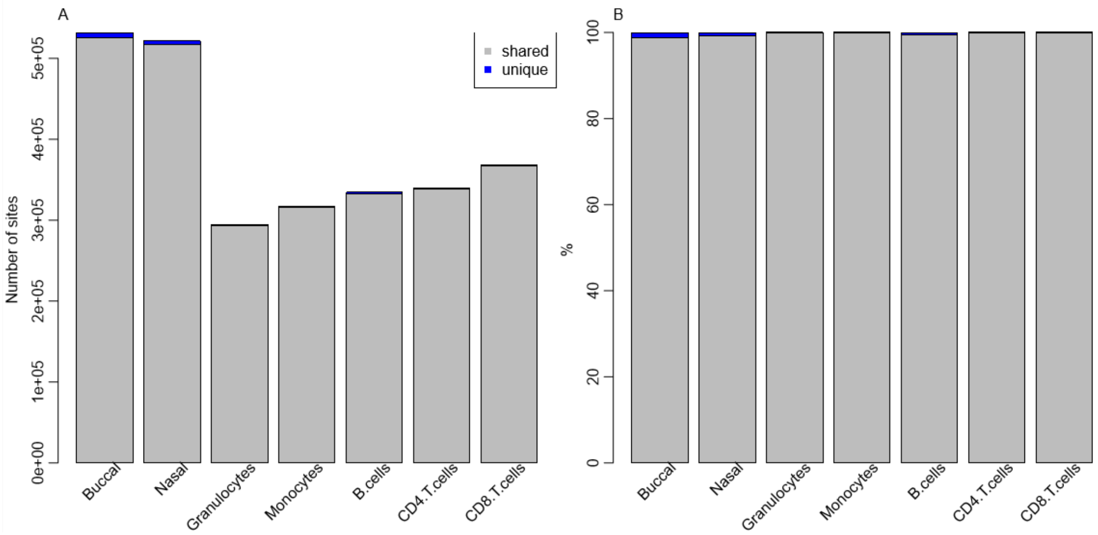

Supplement: S3 Fig — For each sample type the sites identified as differentially methylated relative to whole blood were categorized into those that are uniquely different in that sample type or shared with at least one other sample type. Unique DMPs were defined as those where the t-statistic comparing each sample type to whole blood were significant for only a single sample-type. Bar chart A) shows the number and B) shows the percentage of unique and shared DMPs compared to whole blood for each sample type. (PDF) [file pgen.1009443.s003.pdf]
