# Supplementary material for: Assessing the co-variability of DNA methylation across peripheral cells and tissues: Implications for the interpretation of findings in epigenetic epidemiology
Source: PLoS Genet. 2021 Mar 19;17(3):e1009443. doi: 10.1371/journal.pgen.1009443 (PMC8011804; doi:10.1371/journal.pgen.1009443)

Figure S5. Heatmap showing the overlap between sample-types for all identified differentially methylated positions. Taking all sites identified as having a significantly different level of DNA methylation compared to whole blood in at least one sample type (n = 611,070; ANOVA $P < 9x10^{-8}$) we counted the number each of individual sample types characterized by differential DNAm ($P < 0.05$). Each box in this heatmap represents the percentage of significant DMPs that are shared between two sample types.

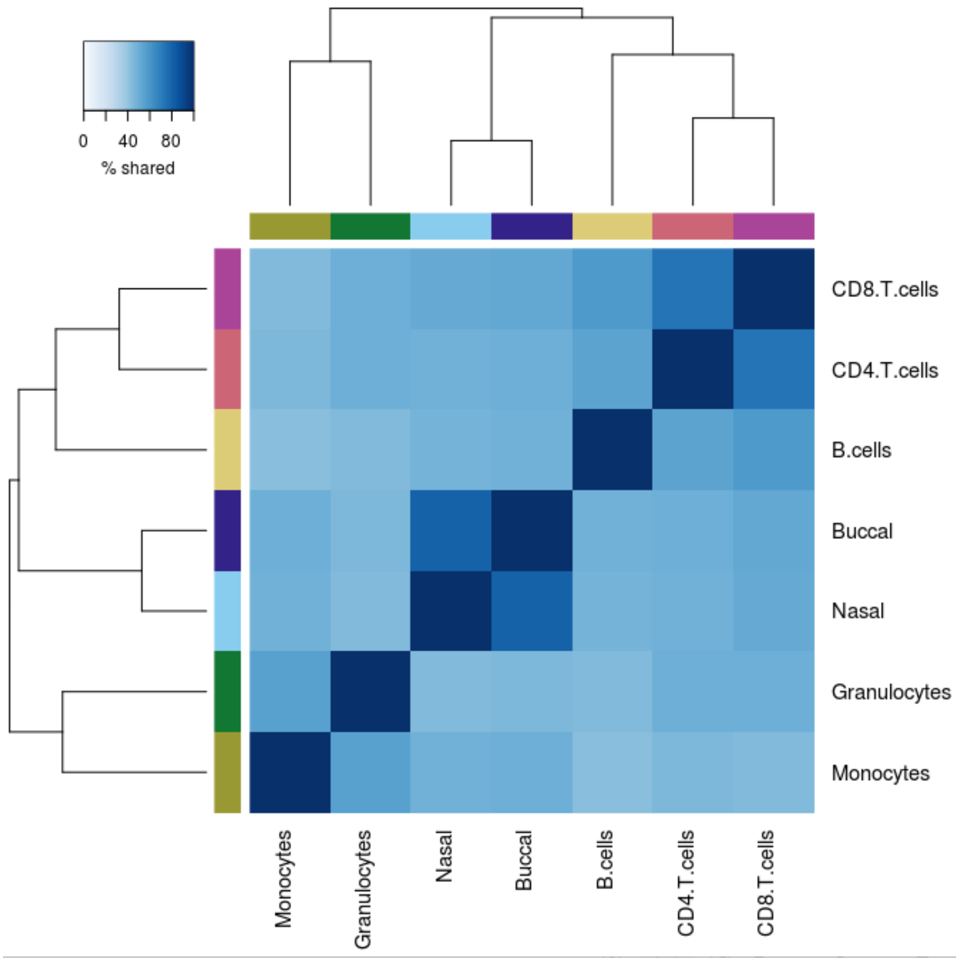

Supplement: S5 Fig — Taking all sites identified as having a significantly different level of DNA methylation compared to whole blood in at least one sample type (n = 611,070; ANOVA P < 9x10^-8^) we counted the number each of individual sample types characterized by differential DNAm (P < 0.05). Each box in this heatmap represents the percentage of significant DMPs that are shared between two sample types. (PDF) [file pgen.1009443.s005.pdf]
