# Supplementary material for: Assessing the co-variability of DNA methylation across peripheral cells and tissues: Implications for the interpretation of findings in epigenetic epidemiology
Source: PLoS Genet. 2021 Mar 19;17(3):e1009443. doi: 10.1371/journal.pgen.1009443 (PMC8011804; doi:10.1371/journal.pgen.1009443)

Figure S6. Histogram showing the most common intersects between sample-types for all differentially methylated positions. Considering all sites identified as having a significantly different level of DNA methylation in at least sample type compared to whole blood (n = 611,070; ANOVA $P < 9 \times 10^{-8}$) we considered t-statistics to identify the specific sample types characterized by differential DNA methylation. Shown are the combinations of sample types with the most shared DMPs, with the vertical histogram at the top indicating the number of shared DMPs and the matrix underneath highlighting specific combinations of sample type. The colored bars in the horizontal histogram in the bottom left indicate the total number of DMPs for each sample type.

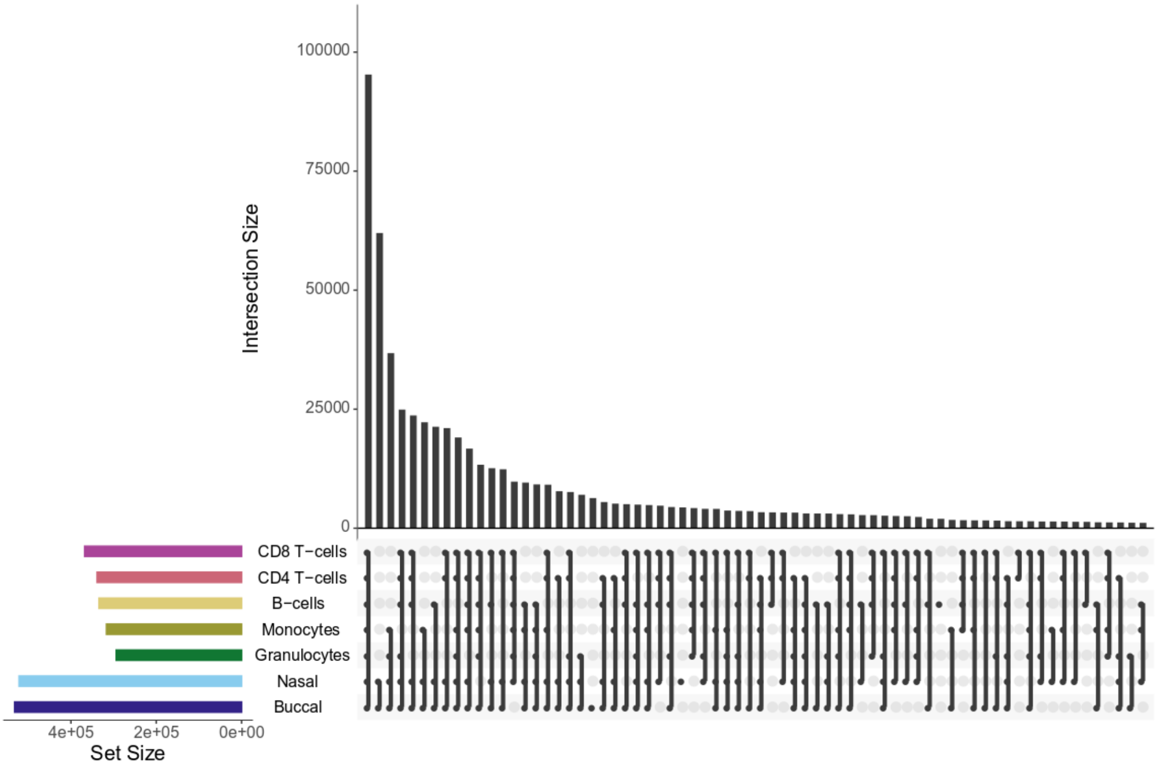

Supplement: S6 Fig — Considering all sites identified as having a significantly different level of DNA methylation in at least sample type compared to whole blood (n = 611,070; ANOVA P < 9x10^-8^) we considered t-statistics to identify the specific sample types characterized by differential DNA methylation. Shown are the combinations of sample types with the most shared DMPs, with the vertical histogram at the top indicating the number of shared DMPs and the matrix underneath highlighting specific combinations of sample type. The colored bars in the horizontal histogram in the bottom left indicate the total number of DMPs for each sample type. (PDF) [file pgen.1009443.s006.pdf]
