# Supplementary material for: Assessing the co-variability of DNA methylation across peripheral cells and tissues: Implications for the interpretation of findings in epigenetic epidemiology
Source: PLoS Genet. 2021 Mar 19;17(3):e1009443. doi: 10.1371/journal.pgen.1009443 (PMC8011804; doi:10.1371/journal.pgen.1009443)

Figure S7. Density plot of the variation in DNA methylation for each sample-type. Shown across all autosomal DNAm sites included in our analysis is the distribution of the standard deviation in DNAm at each site. Each sample-type is represented by a different coloured line. Our results show that in general, DNA methylation measured in buccal (purple) or nasal (blue) epithelial samples is more variable across individuals than DNA methylation measured in whole blood and individual constituent blood cell types.

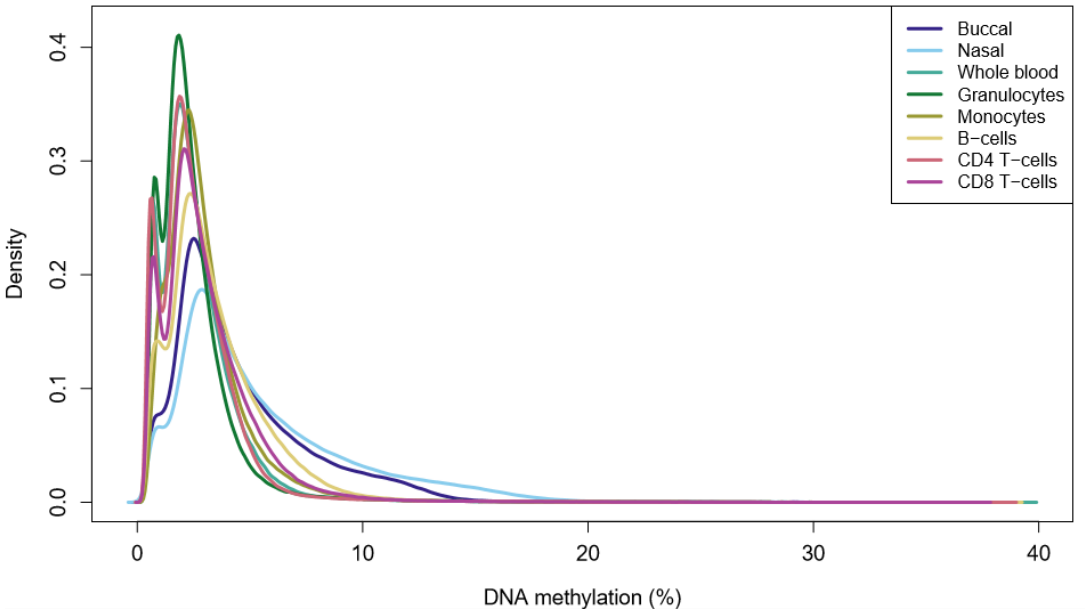

Supplement: S7 Fig — Shown across all autosomal DNAm sites included in our analysis is the distribution of the standard deviation in DNAm at each site. Each sample-type is represented by a different coloured line. Our results show that in general, DNA methylation measured in buccal (purple) or nasal (blue) epithelial samples is more variable across individuals than DNA methylation measured in whole blood and individual constituent blood cell types. (PDF) [file pgen.1009443.s007.pdf]
