# Supplementary material for: Assessing the co-variability of DNA methylation across peripheral cells and tissues: Implications for the interpretation of findings in epigenetic epidemiology
Source: PLoS Genet. 2021 Mar 19;17(3):e1009443. doi: 10.1371/journal.pgen.1009443 (PMC8011804; doi:10.1371/journal.pgen.1009443)

Figure S8. **Scatterplot comparing the site-specific variance in DNA methylation between different sample-types.** Shown is the standard deviation in DNA methylation for all autosomal DNAm sites included in our analysis for each pairwise combination of sample types.

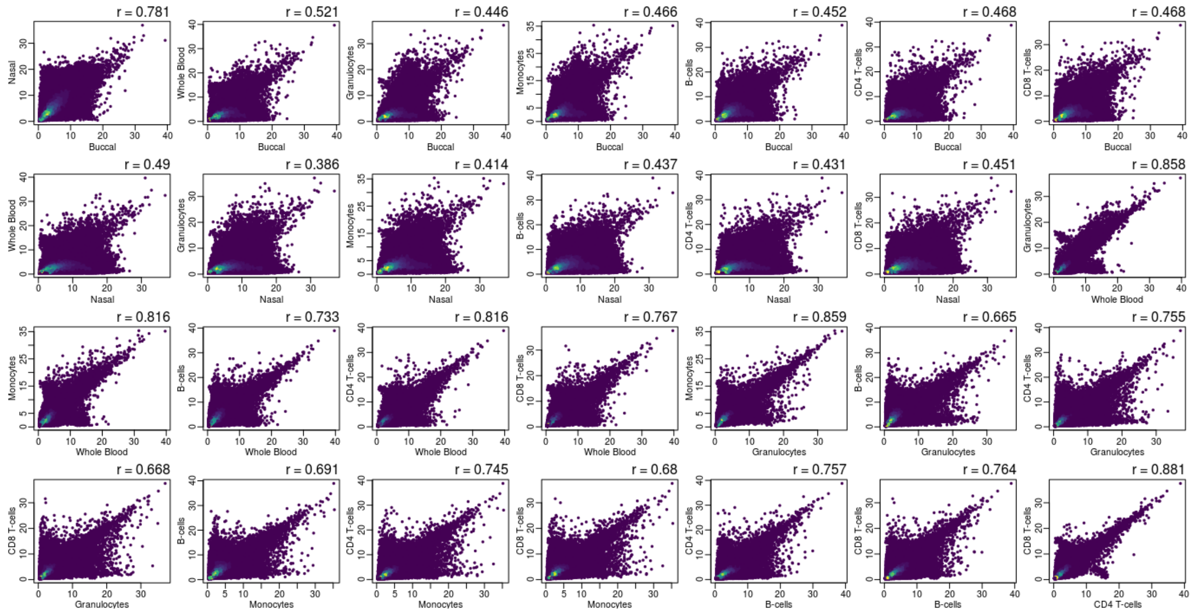

Supplement: S8 Fig — Shown is the standard deviation in DNA methylation for all autosomal DNAm sites included in our analysis for each pairwise combination of sample types. (PDF) [file pgen.1009443.s008.pdf]
