# Supplementary material for: Assessing the co-variability of DNA methylation across peripheral cells and tissues: Implications for the interpretation of findings in epigenetic epidemiology
Source: PLoS Genet. 2021 Mar 19;17(3):e1009443. doi: 10.1371/journal.pgen.1009443 (PMC8011804; doi:10.1371/journal.pgen.1009443)

Figure S9. Density plot of the variation in DNAm for each sample-type for differentially variable sites. Each sample-type is represented by a different colored line. This plot shows that sites with significant variance across sample types are generally characterized by increased variance in buccal (purple) and nasal (blue) epithelial samples compared to whole blood and individual constituent blood cell types.

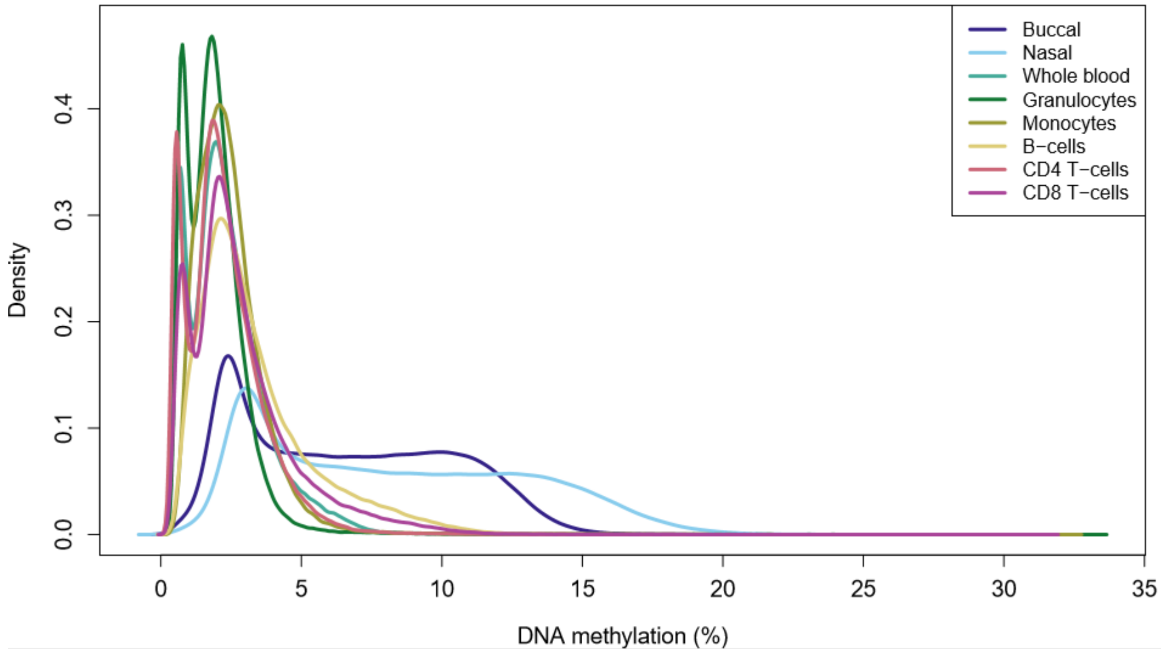

Supplement: S9 Fig — Each sample-type is represented by a different colored line. This plot shows that sites with significant variance across sample types are generally characterized by increased variance in buccal (purple) and nasal (blue) epithelial samples compared to whole blood and individual constituent blood cell types. (PDF) [file pgen.1009443.s009.pdf]
