# Supplementary material for: Assessing the co-variability of DNA methylation across peripheral cells and tissues: Implications for the interpretation of findings in epigenetic epidemiology
Source: PLoS Genet. 2021 Mar 19;17(3):e1009443. doi: 10.1371/journal.pgen.1009443 (PMC8011804; doi:10.1371/journal.pgen.1009443)

Figure S10. Scatterplot of the site-specific variance in DNA methylation between different sample types across DNAm sites with significantly different levels of variation (n = 194, 247). Above each plot is the Pearson correlation coefficient.

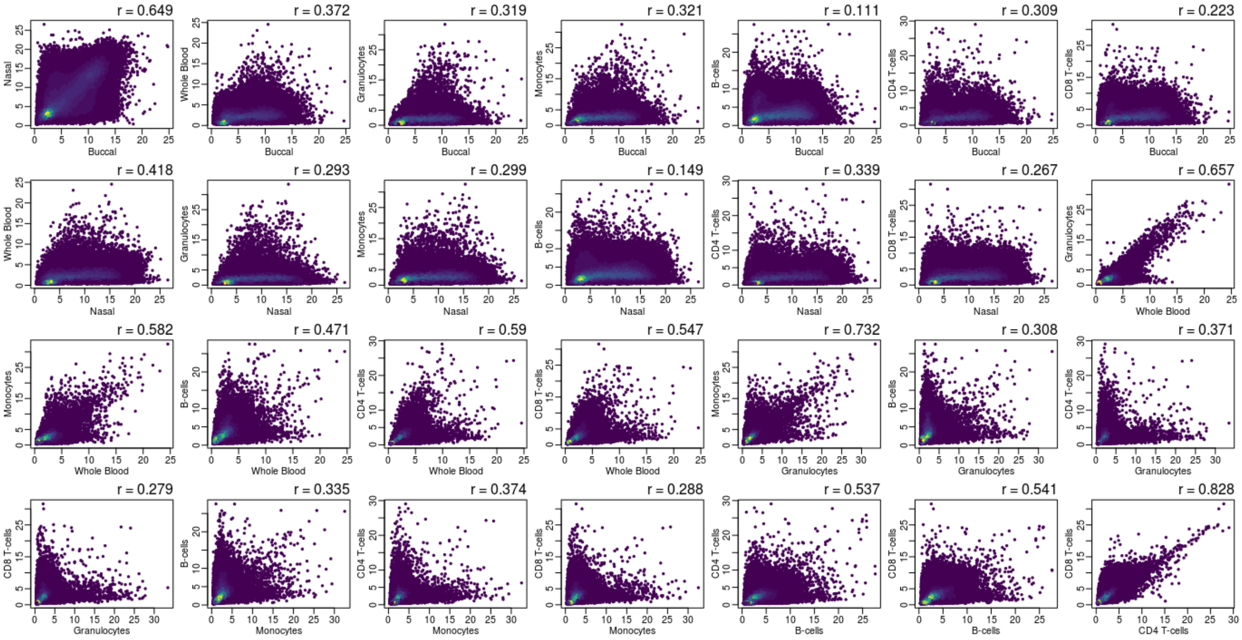

Supplement: S10 Fig — Above each plot is the Pearson correlation coefficient. (PDF) [file pgen.1009443.s010.pdf]
