# Supplementary material for: Assessing the co-variability of DNA methylation across peripheral cells and tissues: Implications for the interpretation of findings in epigenetic epidemiology
Source: PLoS Genet. 2021 Mar 19;17(3):e1009443. doi: 10.1371/journal.pgen.1009443 (PMC8011804; doi:10.1371/journal.pgen.1009443)

Figure S12. Inter-individual variation in DNA methylation in whole blood is correlated with variation in isolated blood cell types. Histograms showing the distribution of correlation coefficients between DNA methylation in whole blood and the five blood cell types. **A)** B-cells, **B)** CD4 T-cells, **C)** CD8 T-cells, **D)** monocytes and **E)** granulocytes. The vertical blue dashed line indicates a correlation coefficient of zero. For all five cell types the distribution of correlation coefficients is skewed to the right.

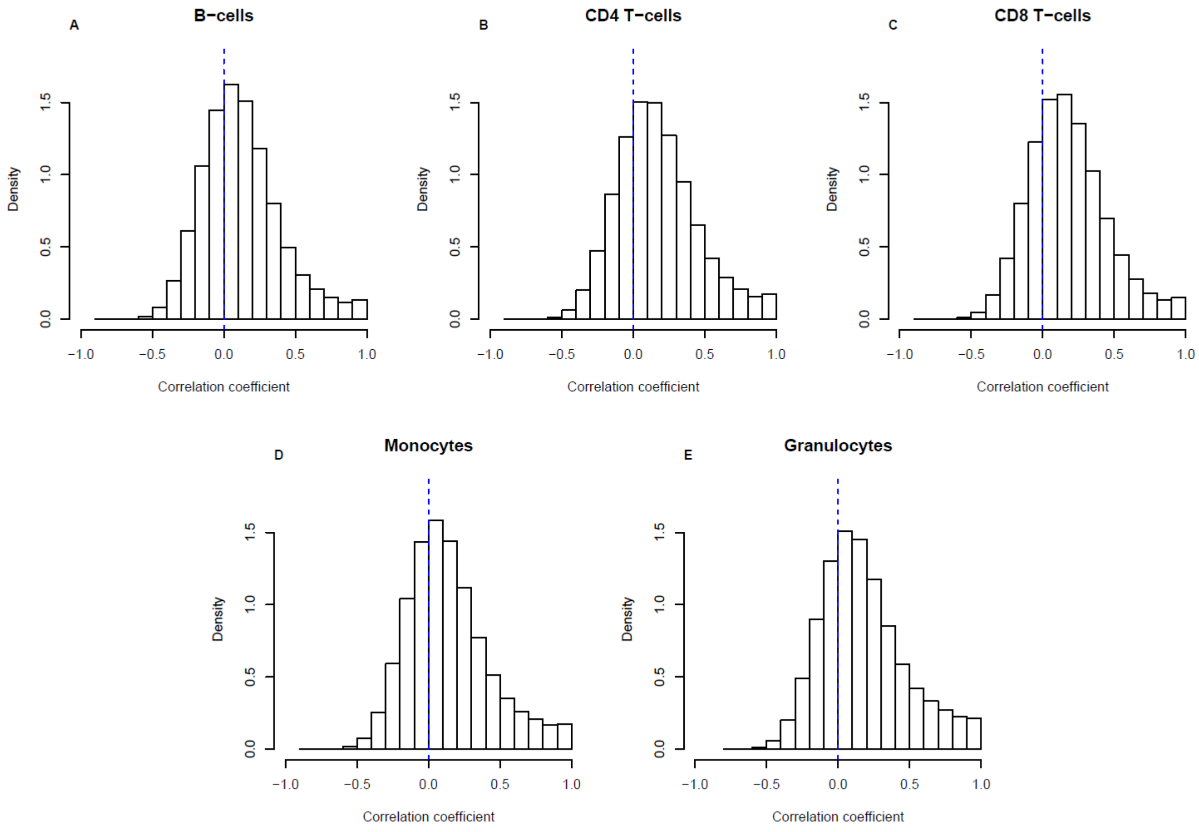

Supplement: S12 Fig — Histograms showing the distribution of correlation coefficients between DNA methylation in whole blood and the five blood cell types. A) B-cells, B) CD4 T-cells, C) CD8 T-cells, D) monocytes and E) granulocytes. The vertical blue dashed line indicates a correlation coefficient of zero. For all five cell types the distribution of correlation coefficients is skewed to the right. (PDF) [file pgen.1009443.s012.pdf]
