# Supplementary material for: Assessing the co-variability of DNA methylation across peripheral cells and tissues: Implications for the interpretation of findings in epigenetic epidemiology
Source: PLoS Genet. 2021 Mar 19;17(3):e1009443. doi: 10.1371/journal.pgen.1009443 (PMC8011804; doi:10.1371/journal.pgen.1009443)

Figure S13. Covariation in DNA methylation between whole blood and individual blood cell-types is higher in the subset of DNAm sites classed as having 'intermediate' levels of DNAm or being highly 'variable'. Shown are boxplots of variance explained in whole blood for each cell type separately where DNAm sites are split by mean DNA methylation level (x-axis, left panels) and variability (right panels).

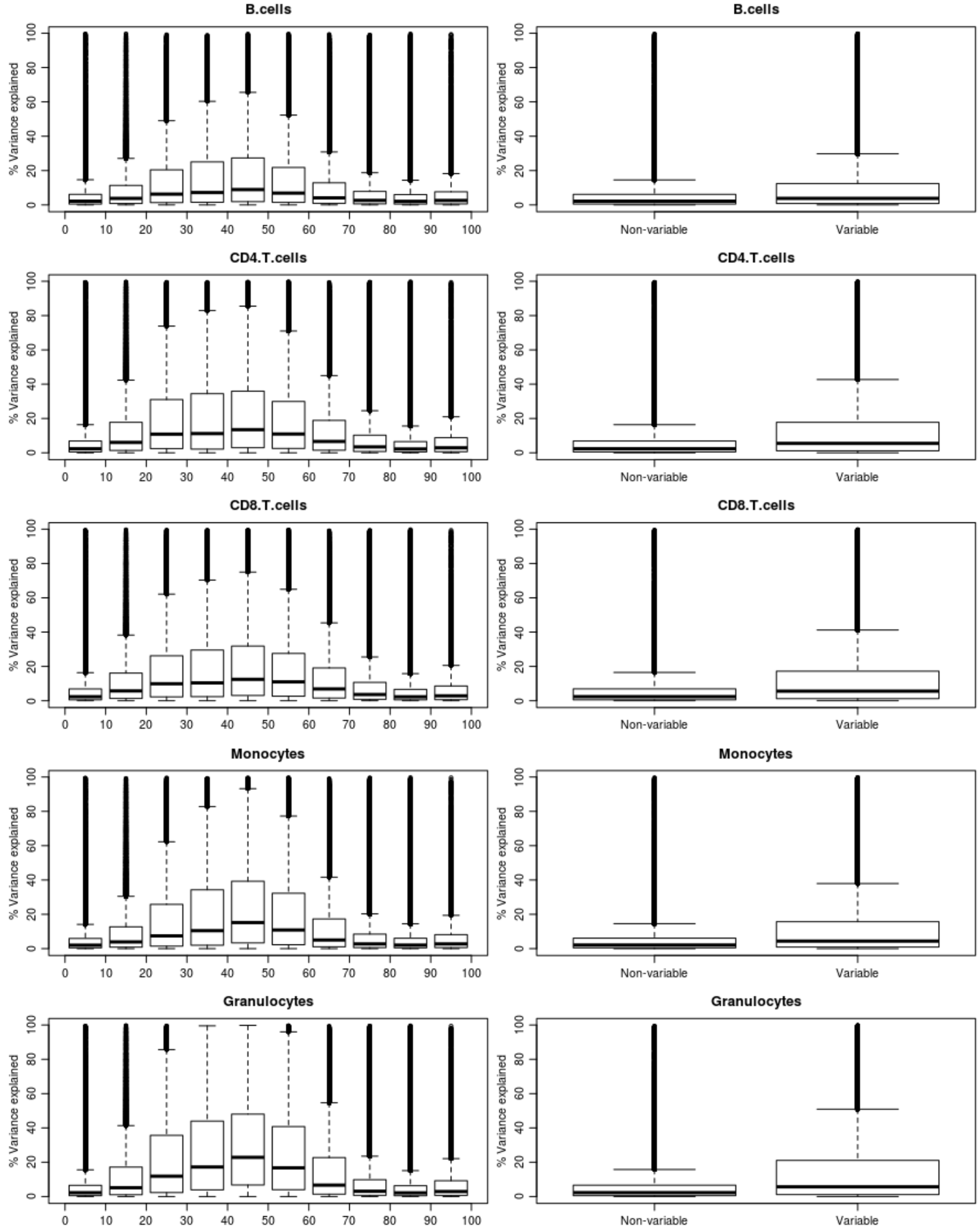

Supplement: S13 Fig — Shown are boxplots of variance explained in whole blood for each cell type separately where DNAm sites are split by mean DNA methylation level (x-axis, left panels) and variability (right panels). (PDF) [file pgen.1009443.s013.pdf]
