# Supplementary material for: Assessing the co-variability of DNA methylation across peripheral cells and tissues: Implications for the interpretation of findings in epigenetic epidemiology
Source: PLoS Genet. 2021 Mar 19;17(3):e1009443. doi: 10.1371/journal.pgen.1009443 (PMC8011804; doi:10.1371/journal.pgen.1009443)
Supplement: S15 Fig — Shown for the subset of sites where whole blood DNAm is under strong genetic control (additive genetic effects on DNAm > 0.80, n = 6,617 sites) using estimates from Hannon et al[25] is the proportion of sites (y-axis) for which variation in whole blood DNA methylation explains different levels of variance in five blood cell types (monocytes, granulocytes, CD4+ T cells, CD8+ T cells and B cells) isolated from the same individuals. (PDF) [file pgen.1009443.s015.pdf]

Figure S15. Variation in DNA methylation in whole blood as a predictor of variation in the isolated blood cell types and other peripheral tissues across sites at which DNAm is under strong genetic control. Shown for the subset of sites where whole blood DNAm is under strong genetic control (additive genetic effects on DNAm > 0.80, n = 6,617 sites) using estimates from Hannon et al[1] is the proportion of sites (y-axis) for which variation in whole blood DNA methylation explains different levels of variance in five blood cell types (monocytes, granulocytes, $CD4^+$ T cells, $CD8^+$ T cells and B cells) isolated from the same individuals.

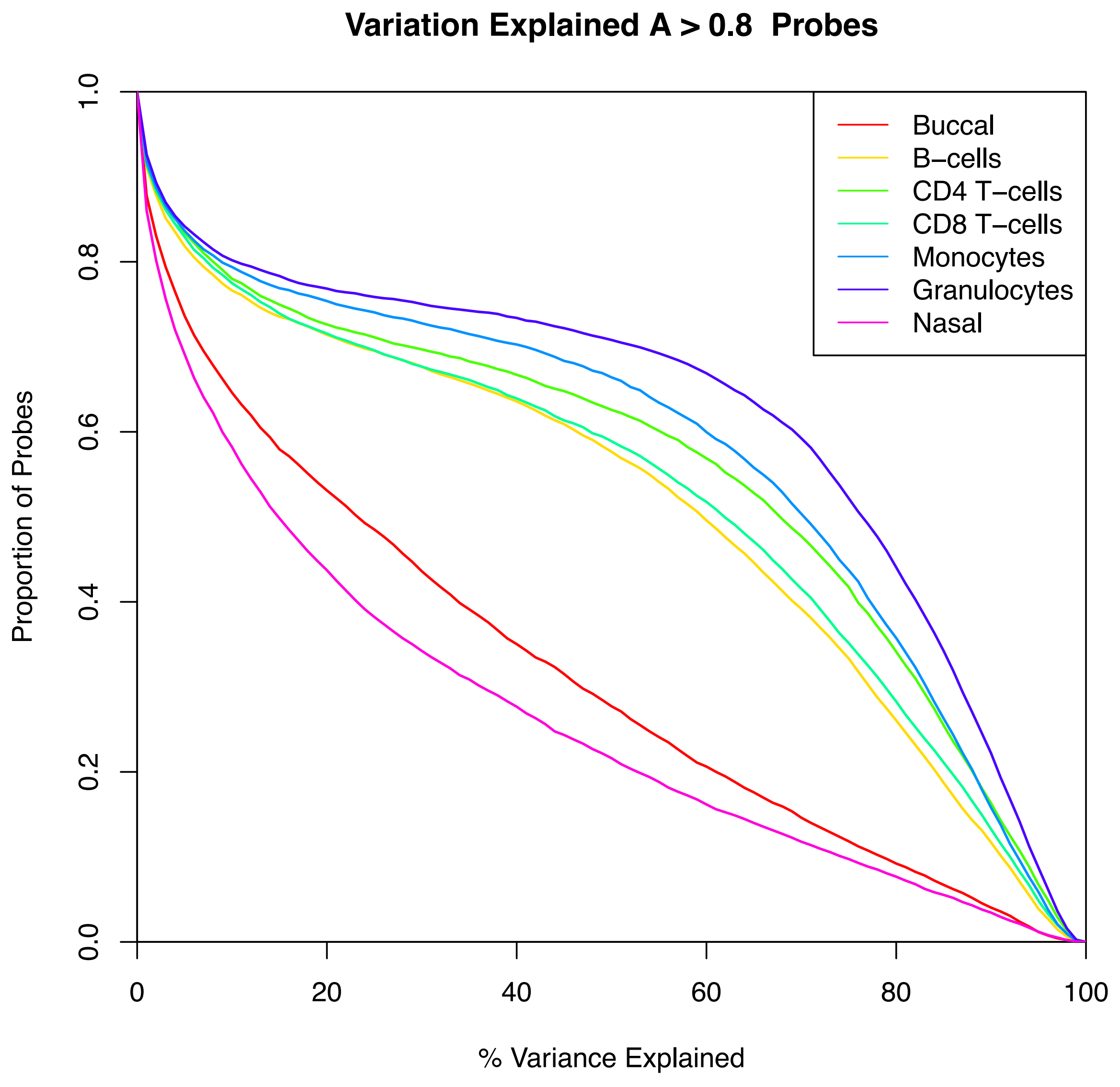

1. Hannon, E., et al., Characterizing genetic and environmental influences on variable DNA methylation using monozygotic and dizygotic twins. PLoS Genet, 2018. 14(8): p. e1007544.
