# Supplementary material for: Assessing the co-variability of DNA methylation across peripheral cells and tissues: Implications for the interpretation of findings in epigenetic epidemiology
Source: PLoS Genet. 2021 Mar 19;17(3):e1009443. doi: 10.1371/journal.pgen.1009443 (PMC8011804; doi:10.1371/journal.pgen.1009443)
Supplement: S16 Fig — Histograms showing the distribution of correlation coefficients between DNA methylation in whole blood and the five blood cell types the subset of sites (n = 147,683 sites) where whole blood DNAm is associated with an mQTL variant using data from Hannon et al[26]. A) B-cells, B) CD4 T-cells, C) CD8 T-cells, D) monocytes and E) granulocytes. The vertical blue dashed line indicates a correlation coefficient of zero. For all five cell types the distribution of correlation coefficients is skewed to the right. (PDF) [file pgen.1009443.s016.pdf]

Figure S16. Inter-individual variation in DNA methylation in whole blood is highly correlated with variation in isolated blood cell types for sites associated with an mQTL variant. Histograms showing the distribution of correlation coefficients between DNA methylation in whole blood and the five blood cell types the subset of sites (n = 147,683 sites) where whole blood DNAm is associated with an mQTL variant using data from Hannon et al[1]. **A)** B-cells, **B)** CD4 T-cells, **C)** CD8 T-cells, **D)** monocytes and **E)** granulocytes. The vertical blue dashed line indicates a correlation coefficient of zero. For all five cell types the distribution of correlation coefficients is skewed to the right.

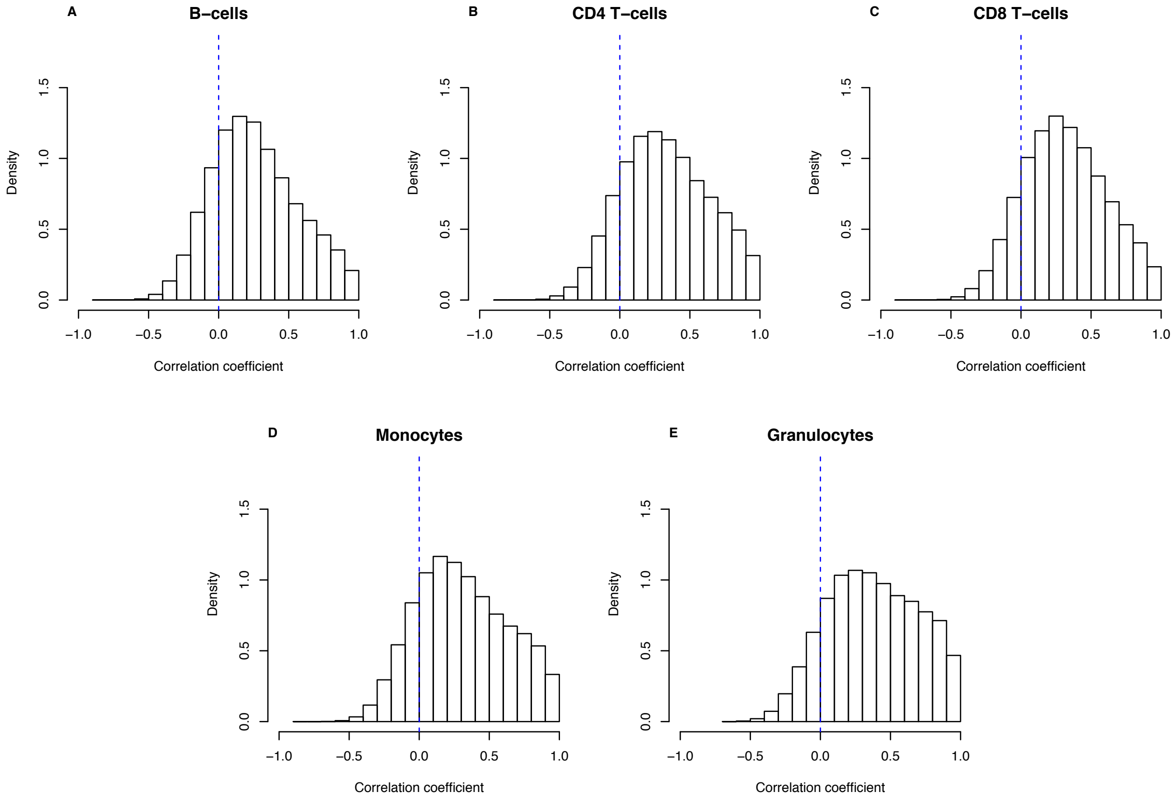

1. Hannon, E., et al., Leveraging DNA-Methylation Quantitative-Trait Loci to Characterize the Relationship between Methylomic Variation, Gene Expression, and Complex Traits. Am J Hum Genet, 2018. 103(5): p. 654-665.
