# Supplementary material for: Assessing the co-variability of DNA methylation across peripheral cells and tissues: Implications for the interpretation of findings in epigenetic epidemiology
Source: PLoS Genet. 2021 Mar 19;17(3):e1009443. doi: 10.1371/journal.pgen.1009443 (PMC8011804; doi:10.1371/journal.pgen.1009443)
Supplement: S17 Fig — Shown for the subset of sites (n = 147,683 sites) where whole blood DNAm is associated with an mQTL variant using data from Hannon et al[26] is the proportion of sites (y-axis) for which variation in whole blood DNA methylation explains different levels of variance in five blood cell types (monocytes, granulocytes, CD4+ T cells, CD8+ T cells and B cells) isolated from the same individuals. (PDF) [file pgen.1009443.s017.pdf]

Figure S17. Variation in DNA methylation in whole blood as a predictor of variation in the isolated blood cell types and other peripheral tissues across sites at which DNAm is associated with an mQTL variant. Shown for the subset of sites (n = 147,683 sites) where whole blood DNAm is associated with an mQTL variant using data from Hannon et al[1] is the proportion of sites (y-axis) for which variation in whole blood DNA methylation explains different levels of variance in five blood cell types (monocytes, granulocytes, $CD4^+$ T cells, $CD8^+$ T cells and B cells) isolated from the same individuals.

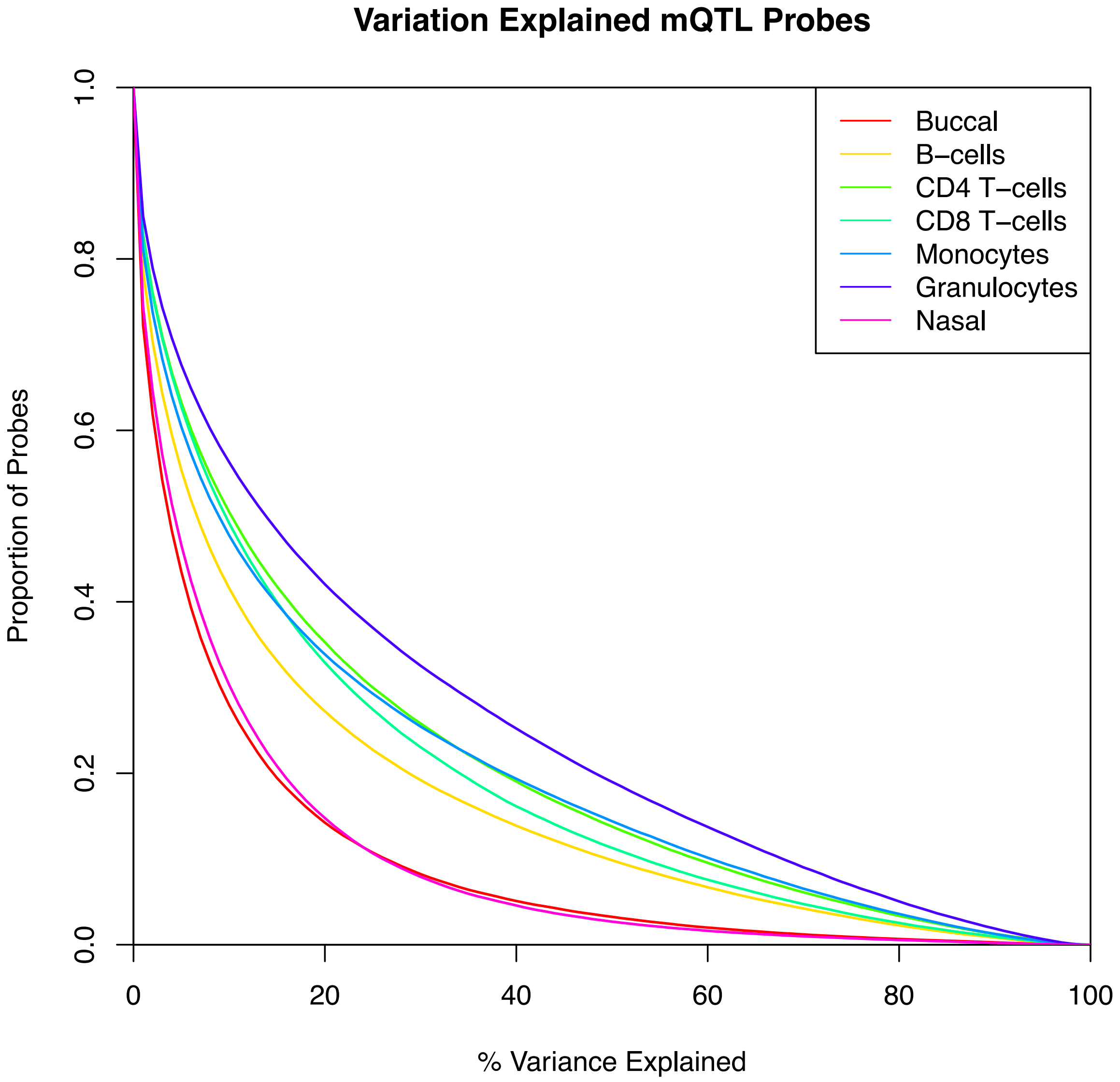

1. Hannon, E., et al., Leveraging DNA-Methylation Quantitative-Trait Loci to Characterize the Relationship between Methylomic Variation, Gene Expression, and Complex Traits. Am J Hum Genet, 2018. 103(5): p. 654-665.
