# Supplementary material for: Assessing the co-variability of DNA methylation across peripheral cells and tissues: Implications for the interpretation of findings in epigenetic epidemiology
Source: PLoS Genet. 2021 Mar 19;17(3):e1009443. doi: 10.1371/journal.pgen.1009443 (PMC8011804; doi:10.1371/journal.pgen.1009443)
Supplement: S18 Fig — Histograms showing the distribution of correlation coefficients between DNA methylation in whole blood and the five blood cell types the subset of sites where whole blood DNAm is strongly influenced by non-shared environmental factors (non-shared environmental effects on DNAm > 0.80, n = 143,501 sites) using estimates from Hannon et al[25]. A) B-cells, B) CD4 T-cells, C) CD8 T-cells, D) monocytes and E) granulocytes. The vertical blue dashed line indicates a correlation coefficient of zero. (PDF) [file pgen.1009443.s018.pdf]

Figure S18. Inter-individual variation in DNA methylation in whole blood is highly correlated with variation in isolated blood cell types for sites strongly influenced by non-shared environmental factors. Histograms showing the distribution of correlation coefficients between DNA methylation in whole blood and the five blood cell types the subset of sites where whole blood DNAm is strongly influenced by non-shared environmental factors (non-shared environmental effects on DNAm > 0.80, n = 143,501 sites) using estimates from Hannon et al[1]. **A)** B-cells, **B)** CD4 T-cells, **C)** CD8 T-cells, **D)** monocytes and **E)** granulocytes. The vertical blue dashed line indicates a correlation coefficient of zero.

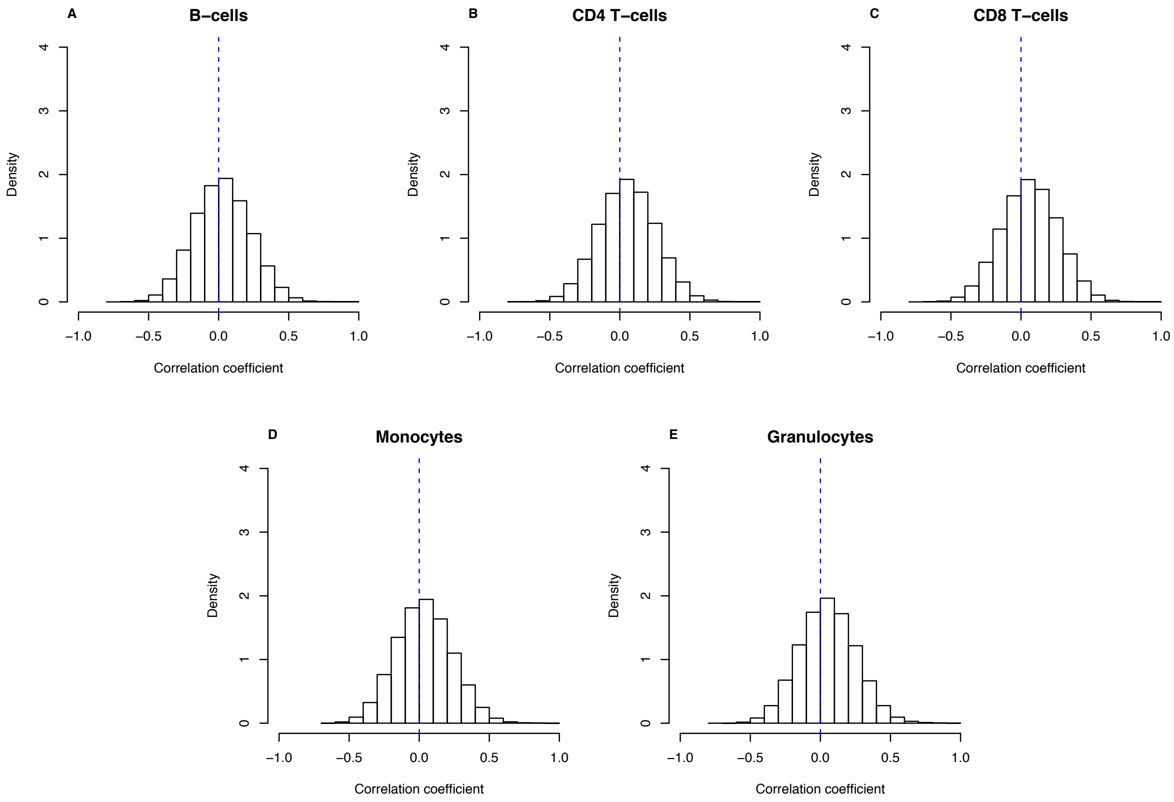

1. Hannon, E., et al., Characterizing genetic and environmental influences on variable DNA methylation using monozygotic and dizygotic twins. PLoS Genet, 2018. 14(8): p. e1007544.
