# Supplementary material for: Assessing the co-variability of DNA methylation across peripheral cells and tissues: Implications for the interpretation of findings in epigenetic epidemiology
Source: PLoS Genet. 2021 Mar 19;17(3):e1009443. doi: 10.1371/journal.pgen.1009443 (PMC8011804; doi:10.1371/journal.pgen.1009443)
Supplement: S19 Fig — Shown for the subset of sites where whole blood DNAm is strongly influenced by non-shared environmental factors (non-shared environmental effects on DNAm > 0.80, n = 143,501 sites) using estimates from Hannon et al[25] is the proportion of sites (y-axis) for which variation in whole blood DNA methylation explains different levels of variance in five blood cell types (monocytes, granulocytes, CD4+ T cells, CD8+ T cells and B cells) isolated from the same individuals. (PDF) [file pgen.1009443.s019.pdf]

Figure S19. Variation in DNA methylation in whole blood as a predictor of variation in the isolated blood cell types and other peripheral tissues for sites strongly influenced by non-shared environmental factors. Shown for the subset of sites where whole blood DNAm is strongly influenced by non-shared environmental factors (non-shared environmental effects on DNAm > 0.80, n = 143,501 sites) using estimates from Hannon et al[1] is the proportion of sites (y-axis) for which variation in whole blood DNA methylation explains different levels of variance in five blood cell types (monocytes, granulocytes, CD4$^+$ T cells, CD8$^+$ T cells and B cells) isolated from the same individuals.

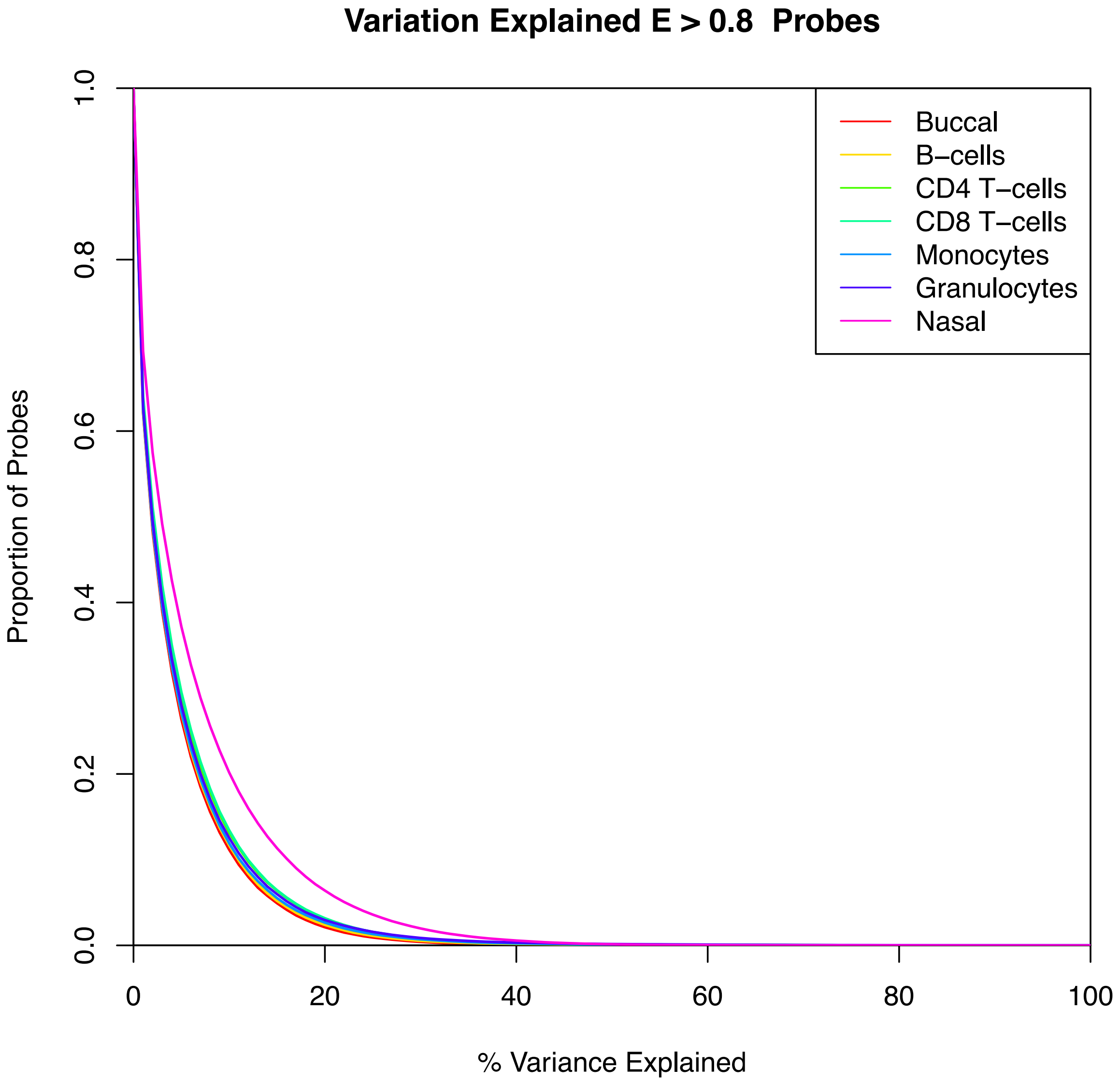

1. Hannon, E., et al., Characterizing genetic and environmental influences on variable DNA methylation using monozygotic and dizygotic twins. PLoS Genet, 2018. 14(8): p. e1007544.
