# Supplementary material for: Assessing the co-variability of DNA methylation across peripheral cells and tissues: Implications for the interpretation of findings in epigenetic epidemiology
Source: PLoS Genet. 2021 Mar 19;17(3):e1009443. doi: 10.1371/journal.pgen.1009443 (PMC8011804; doi:10.1371/journal.pgen.1009443)

Figure S20. Inter-individual variation in different blood cell types predicts inter-individual variation in whole blood at the same sites. Scatterplots comparing blood-cell type correlations between cell types. The colour of the point indicates the density of observations at that position ranging from gray (low) to yellow (high).

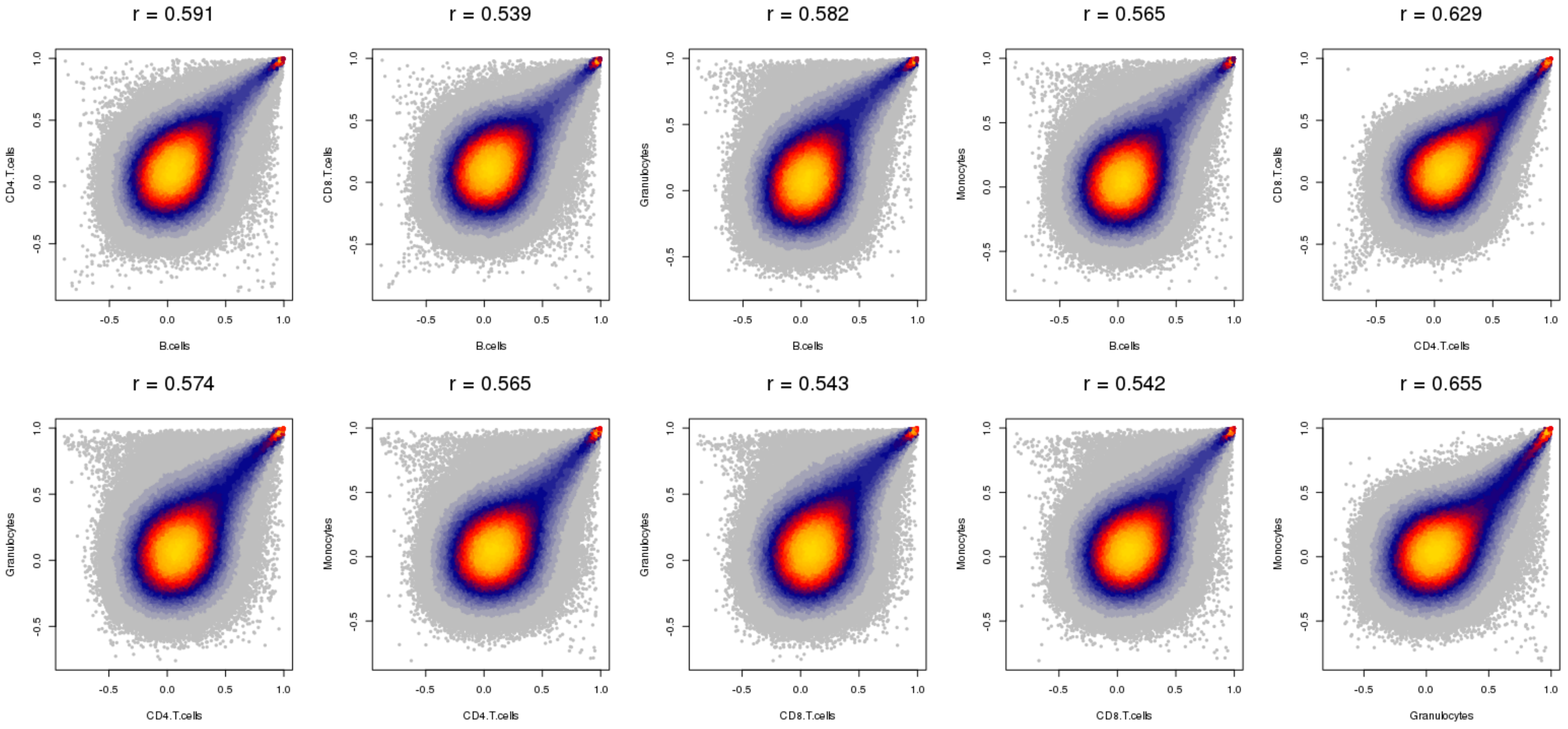

Supplement: S20 Fig — Scatterplots comparing blood-cell type correlations between cell types. The colour of the point indicates the density of observations at that position ranging from gray (low) to yellow (high). (PDF) [file pgen.1009443.s020.pdf]
