# Supplementary figures and images for: Assessing the co-variability of DNA methylation across peripheral cells and tissues: Implications for the interpretation of findings in epigenetic epidemiology
Source: PLoS Genet. 2021 Mar 19;17(3):e1009443. doi: 10.1371/journal.pgen.1009443 (PMC8011804; doi:10.1371/journal.pgen.1009443)

**Figure S21. Histogram of variance explained in whole blood by all five cell types combined.**

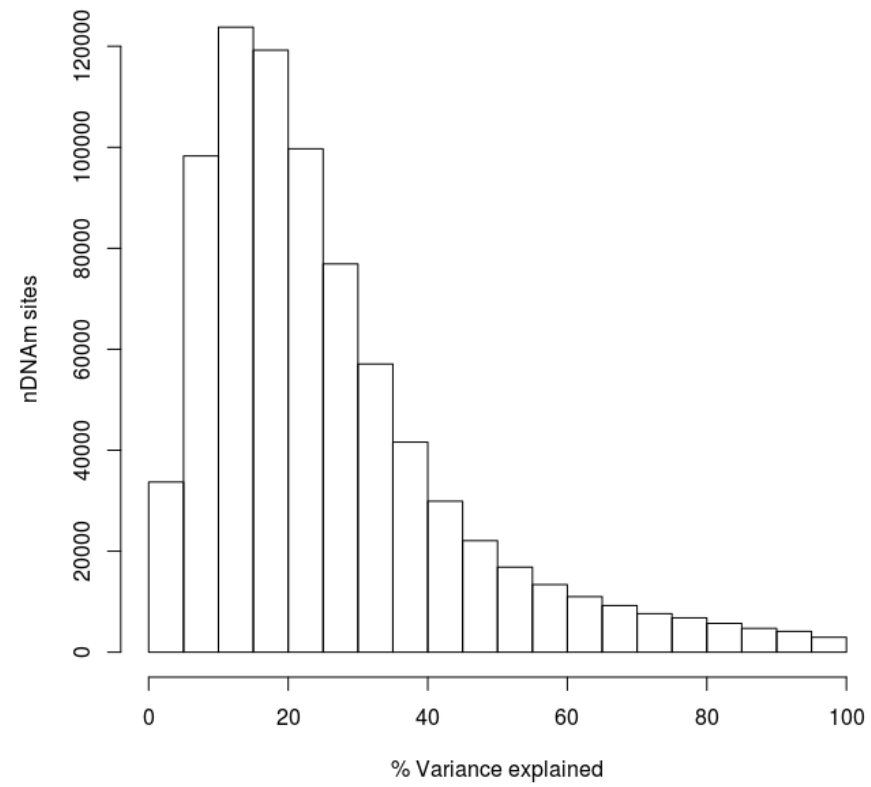

Supplement: S21 Fig — (PDF) [file pgen.1009443.s021.pdf]
