# Supplementary material for: Assessing the co-variability of DNA methylation across peripheral cells and tissues: Implications for the interpretation of findings in epigenetic epidemiology
Source: PLoS Genet. 2021 Mar 19;17(3):e1009443. doi: 10.1371/journal.pgen.1009443 (PMC8011804; doi:10.1371/journal.pgen.1009443)

Figure S22. Boxplots of variance in DNA methylation explained in whole blood by all five cell types combined stratified by A) mean DNA methylation level and B) the variability in DNA methylation at that site.

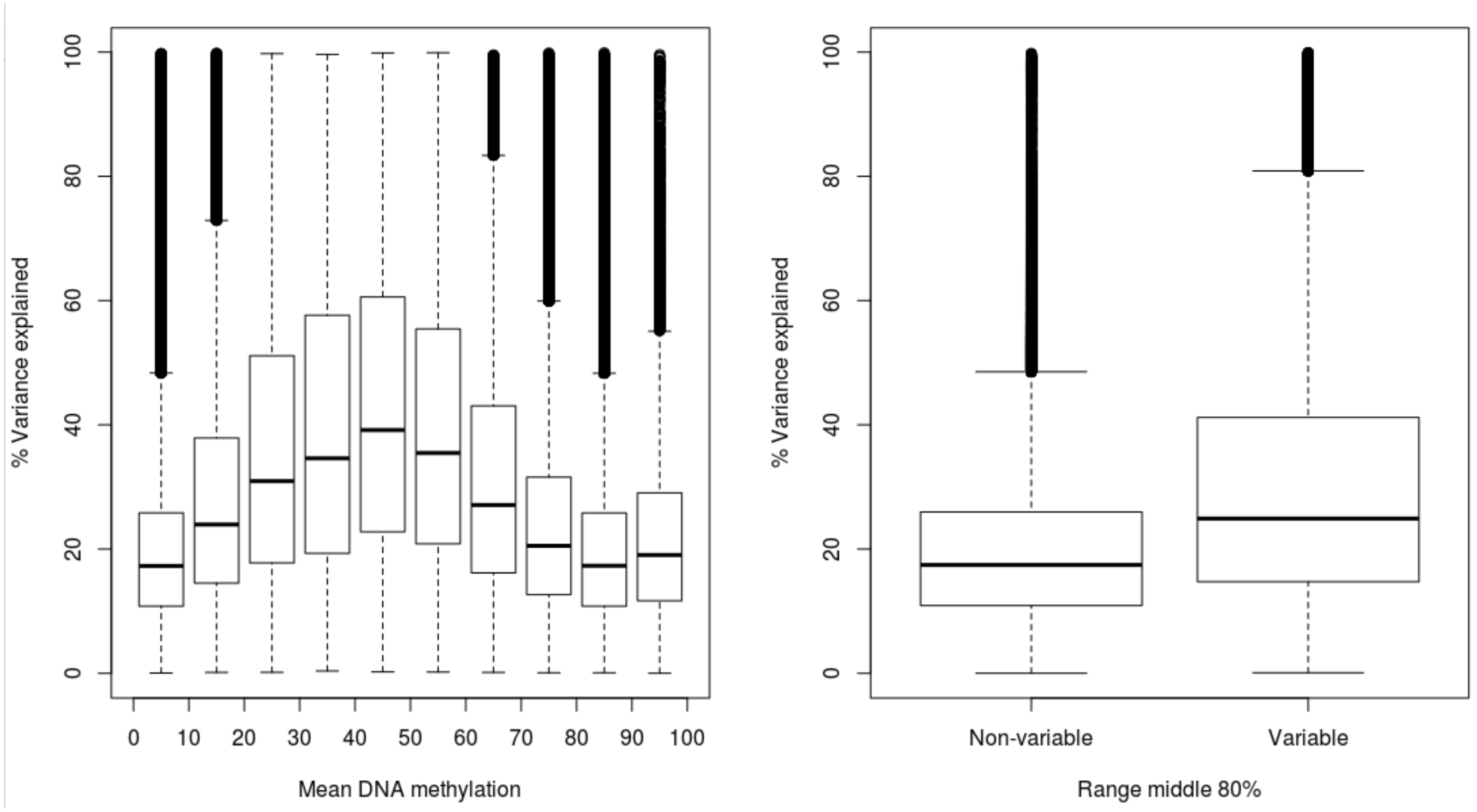

Supplement: S22 Fig — Boxplots of variance in DNA methylation explained in whole blood by all five cell types combined stratified by A) mean DNA methylation level and B) the variability in DNA methylation at that site. (PDF) [file pgen.1009443.s022.pdf]
