# Supplementary material for: Assessing the co-variability of DNA methylation across peripheral cells and tissues: Implications for the interpretation of findings in epigenetic epidemiology
Source: PLoS Genet. 2021 Mar 19;17(3):e1009443. doi: 10.1371/journal.pgen.1009443 (PMC8011804; doi:10.1371/journal.pgen.1009443)

Figure S23. Histogram showing the number of individual blood cell types explaining at least 20% of the variance in DNA methylation in whole blood.

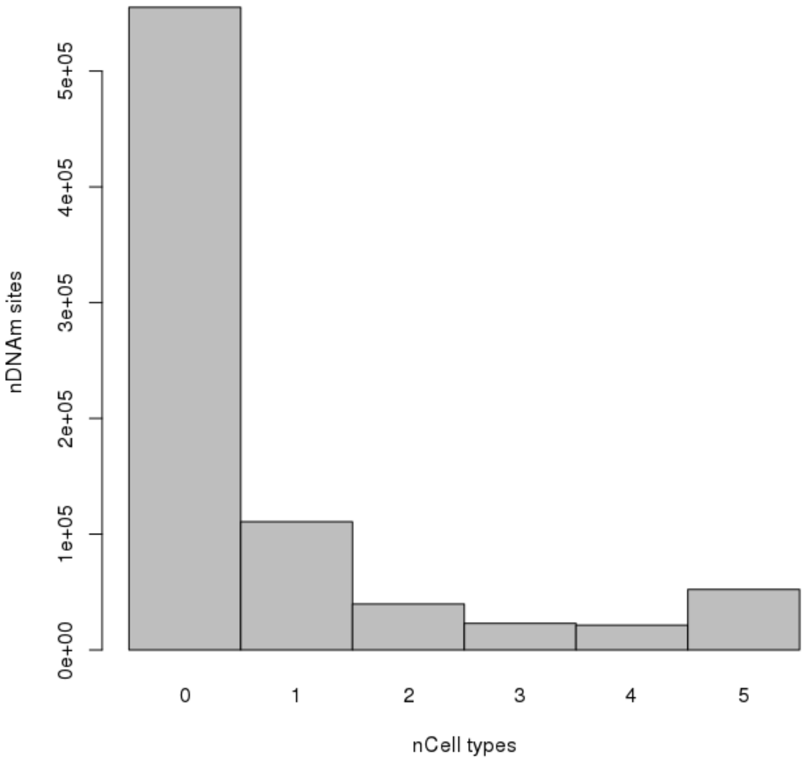

Supplement: S23 Fig — (PDF) [file pgen.1009443.s023.pdf]
