# Supplementary material for: Assessing the co-variability of DNA methylation across peripheral cells and tissues: Implications for the interpretation of findings in epigenetic epidemiology
Source: PLoS Genet. 2021 Mar 19;17(3):e1009443. doi: 10.1371/journal.pgen.1009443 (PMC8011804; doi:10.1371/journal.pgen.1009443)

Figure S24. Heatmap showing the ratio of observed to expected number of characteristic sites for each blood cell type across DMPs associated with a range of traits. Association results were downloaded from the EWAS catalog (http://ewascatalog.org/) and filtered to those identified in whole blood at a significance threshold of P < 1e-7. This heatmap contains all traits characterized by at least five significant associations that were characteristic of a blood cell type. Grey indicates that there was no characteristic sites for that cell type for that trait.

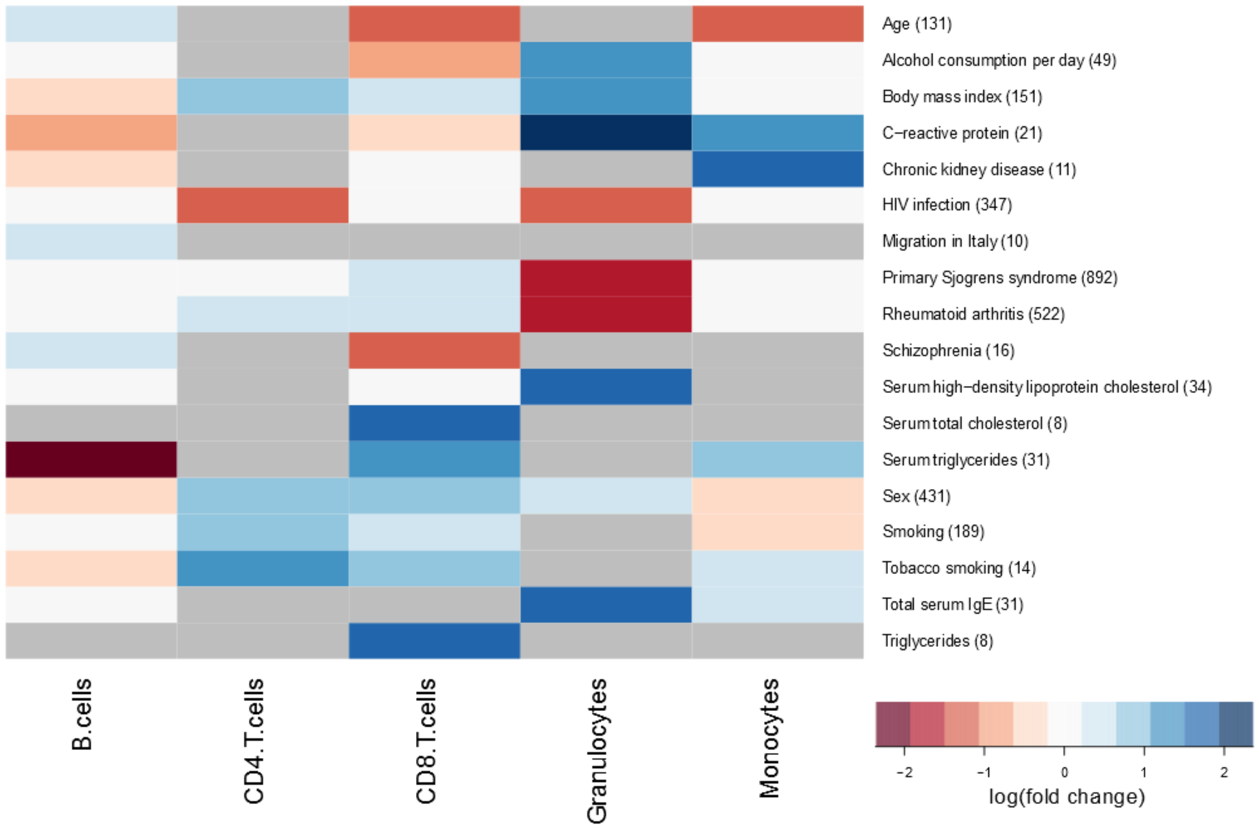

Supplement: S24 Fig — Association results were downloaded from the EWAS catalog (http://ewascatalog.org/) and filtered to those identified in whole blood at a significance threshold of P < 1e-7. This heatmap contains all traits characterized by at least five significant associations that were characteristic of a blood cell type. Grey indicates that there was no characteristic sites for that cell type for that trait. (PDF) [file pgen.1009443.s024.pdf]
