# Supplementary material for: Assessing the co-variability of DNA methylation across peripheral cells and tissues: Implications for the interpretation of findings in epigenetic epidemiology
Source: PLoS Genet. 2021 Mar 19;17(3):e1009443. doi: 10.1371/journal.pgen.1009443 (PMC8011804; doi:10.1371/journal.pgen.1009443)
Supplement: Attachment — Submitted filename: ResponseToReviewers.pdf [file pgen.1009443.s033.pdf]

Manuscript ID: PGENETICS-D-20-00987

We are delighted that the three Reviewers think our study is of interest to the readers of *PLoS Genetics*. Reviewer 1 found our manuscript interesting and commended us for already making our data publicly available through GEO. Reviewer 3 found our study to be of broad interest, concluding it was "…*well-written and its conclusions carefully considered*…"; they appreciated "…*the note of caution it introduces to the interpretation of EWAS studies*…".

We thank all three Reviewers for their constructive comments and suggestions; we have considered these in detail and made significant improvements to the manuscript as a result. Responses to individual comments by the three Reviewers are listed below. We recognise that the original manuscript was quite dense and some of the key messages were not easily accessible to the Reviewers. We hope you will find our revised manuscript much improved and acceptable for publication in *PLoS Genetics*.

Reviewer 1:

1) ***The Reviewer asks for clarification on whether our analyses were based on beta-values or M-values, raising concerns about potential heteroscedasticity with beta-values.***

Our analyses were based on estimates of DNA methylation (DNAm) measured as beta values. We apologize if this was not clear in the original manuscript and have now made this more explicit. We have added a paragraph to the Discussion justifying our use of beta-values and recognising that our focus on beta-values may limit the applicability of our findings to the relatively small number of EWAS datasets where M values are used.

The majority of groups working in epigenetic epidemiology routinely perform association analyses using beta values, rather than M-values, and we believe them to be optimal for the interpretation of differences associated with disease, traits or exposures. Our focus on beta-values, therefore, means that our results will be of maximum utility to groups undertaking EWAS analyses.

We have a strong understanding of the statistical differences between beta-values and M-values. Of note, we have undertaken extensive analyses exploring the potential effect of heteroscedasticity on linear regression models fitted using beta-values, finding that this bias does not lead to an excess of false positives[1]. Importantly, when testing for differences in the level of variation in DNAm between cell-/tissue- types, heteroscedasticity is actually something that we are interested in. In this situation, we do not see heteroscedasticity as a statistical concern; indeed the test we used (Levene's test) is commonly applied to detect heteroscedasticity. The Reviewer is correct in suggesting that when the mean beta-value is substantially different between tissue-/cell-types then the variance may also be, because the variance in beta-values is correlated with the mean. However, this reflects biology; when a DNAm site is either completed methylated or unmethylated within a cell type the level of DNAm is unlikely to vary. In contrast, if the level of DNAm is intermediate, the site is more susceptible to variation in response or to some environmental, genetic or stochastic factor, for example.

A concern with transforming to M-values is that by expanding the range of DNA methylation values at the extremes - so that they are no longer bounded, and the magnitude of variance not constrained - we will no longer be able to detect this type of situation. In addition, we are concerned that the use of M-values is likely to exaggerate technical noise, which is most prevalent at the extreme ends of the distribution of DNAm levels, consequently introducing false positives.

2) ***The Reviewer asks us to add some specifics to the Discussion about the implications of our findings for epigenome-wide association studies.***

We agree with the Reviewer that the implications for EWAS are probably the key utility of the analyses presented in our manuscript. We have therefore expanded our Discussion to include more specific implications for the interpretation of existing EWAS datasets and the implementation of future EWAS studies.

3) ***The Reviewer questions our choice of regression model given that our cohort is derived from twins and suggests with use a mixed effects model instead.***

On reflection, we agree with the Reviewer (and also Reviewer 3 who makes a similar comment) and accept that our choice of regression model to detect differences in DNA methylation between cell/tissue types was incomplete in our original submission. As suggested by the Reviewer we have repeated our analysis using a mixed effects model with nested random effects for family and individual to account for the hierarchical structure of our data. This approach in fact gives us more power and leads to the identification of more differentially methylated positions (DMPs) between sample-types. Our previous linear regression model identified 594,056 DMPs between tissue/cell types, while the mixed effects model identified 611,070 DMPs. The results from the two models are, however, very similar with 593,982 DMPs identified in common using both models and a very strong correlation in p-values between the two models (**Figure 1**; r = 0.863). Of note, a very small number of sites were associated with significant random effects for either family (n = 7,460 sites) or individual (n = 4,033), suggesting that in the context of tissue and cell-type differences, the contribution of genetic or shared environmental effects are minimal. We have additionally rerun the linear model to estimate the proportion of variance in whole blood that is explained by the five purified blood cell types as a mixed effects model. Again, the results between the two regression models were highly correlated (r = 0.961), with the mean total variance explained slightly reduced to 25.7% from 28.0%. We have updated the Results in the manuscript, so that they are based now based on the multilevel analytical model and we thank the Reviewer for this excellent suggestion.

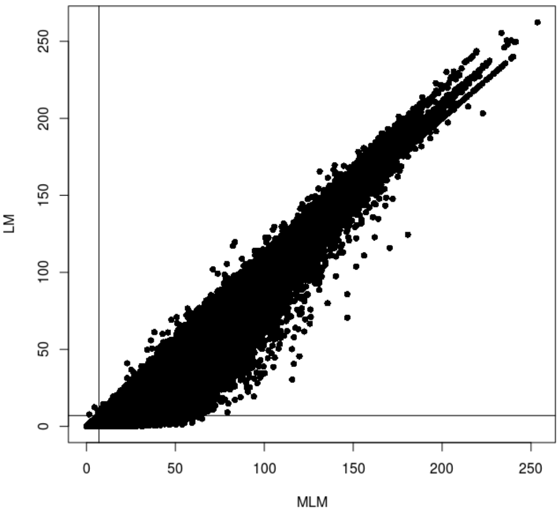

Figure 1 Scatterplot comparing –log10(P-values) for tissue/cell-type differences in DNAm from our original linear regression model (LM; y-axis) and the mixed effects regression model used in our revised manuscript (MLM; x-axis).

4) ***The Reviewer requests we add a table summarising the percentage of cell types from the flow sorting experiment, as well as a summary of the other epidemiological characteristics of the sample.***

We agree with the Reviewer that additional information on the sorting of individual cell types from whole blood would be of interest to readers and have added a Supplementary Table containing this information. Rather than percentages, however, we believe that the absolute

number of cells collected using FACS is a more reliable metric in this context, as the flow sorting of each blood cell type was not performed to completion but rather until a sufficient number of cells was obtained for either the isolation of genomic DNA from that specific fraction or for the cells to be subjected to a subsequent sorting step. Therefore, we caution against the conversion of these numbers into percentages/proportions as they do not necessarily reflect the underlying cellular composition of the whole blood sample from which they were derived. Much of the demographic information requested by the Reviewer (e.g. sex ratios of the twin pairs and summary of smoking pack years) was already provided in the original submission, but as requested we have updated the Supplementary Table containing this information to ensure it is complete.

5) ***The Reviewer was surprised by the number of DMPs (~75% of DNAm sites tested) that we found to be associated with tissue/cell-type differences. They suggest we run sensitivity analyses to gauge the validity of our findings.***

In contrast to the Reviewer, we were not at all surprised by the number of sites detected as being differentially methylated between at least two tissue/cell types! These findings reflect other studies by us and others on blood and other tissue-/cell-types and highlight the major problem of using bulk tissues (such as whole blood) for DNAm analyses in population-based studies. As part of an ongoing (unpublished) analysis of human brain, for example, we have purified nuclei from three neural cell populations (neuronal, oligodendrocytes and microglial) and profiled these using the Illumina EPIC array. Performing a similar analysis to that reported in this manuscript, we identify 546,471 DMPs (73.3% of those tested) that are significantly different between neural cell types. There are also a number of other published studies which identify a similar number of associations. For example, 568,765 DMPs (65.6% of sites tested) were identified in a cohort of 12 females comparing bone and blood profiles [2] and 333,061 DMPs (73% of those tested) were identified when comparing umbilical cord blood white blood cells against placental samples (n = 25 individuals)[3]. Indeed Reviewer 2 (Comment 3) also highlights a number of studies reporting that DNAm profiles in blood cell-types are highly distinctive, exhibiting differences across more sites when compared to other tissues [4, 5]. While some previous studies that have reported fewer significant differences between tissues, these analyses have typically only focused on sites with large differences in DNAm between tissues/cells (e.g. several studies only report sites with a DNAm difference >20%) or have focused on detecting *regions* of differential methylation rather than identifying individual sites (and have therefore reported lower numbers) [6, 7]. Overall, we are confident that our results are reliable and in line with other similar studies; therefore, we do not think sensitivity analyses are warranted, especially given the large numbers of analyses and comparisons already presented in our manuscript.

6) ***Given our findings on the tissue-specificity of the pan-tissue clock the Reviewer suggests that we highlight in the discussion that there are other clocks developed for specific tissues that may lead to more accurate predictions.***

This is a very valid point that we entirely agree with. As requested, we have added some text to the discussion to make this point.

7) ***The Reviewer raises a number of additional queries related to our analysis of differential variation across sample types, including:***

i) Clarification of the specific recommendations result from this analysis for EWAS.

Our rationale for comparing variances in DNAm between samples was to improve understanding of the differences between cell-/tissue-types. Most existing studies only consider differences in the absolute level of DNA methylation when characterising tissue

specific differences, but arguably the differences in the variance of DNAm is more interesting and relevant for epigenetic epidemiology studies. If a site does not vary across a dataset, then there is no reason to study it epidemiologically. If the extent of variation across tissue and cell types varies, then that suggests that the factors that underlie that variation differ, either in their magnitude of effect or the specific combination of associated factors. Therefore, by profiling how variation differs across cell/tissue types, we can gain insight into how epigenetic variation is regulated, and how to interpret associations identified in bulk tissue (i.e. whole blood). This sort of insight is not possible from simply comparing the mean level of DNAm. As in our response to Comment 1 we disagree that M-values should be used as the basis for this analysis, especially as the M-value transformation acts to inflate variation at extreme levels of DNAm.

ii) Concerns that these results are driven by technical variation.

We carefully designed the collection and experimental processing of the samples to minimise any technical variation and we are confident that these effects make a negligible effect on our results. For example, all tissue samples (whole blood, buccal and nasal samples) from both members of each twin-pair were collected at exactly the same time, and blood cell-sorting was performed immediately. All DNA samples were isolated in parallel. We ensured that the samples from each individual and twin pair were profiled in the same experimental batch (see also response to Comment 1ii from Reviewer 3).

As the Reviewer correctly summarizes, DNA methylation sites which are completely methylated or unmethylated across all samples are not affected by biological variation but may be affected by technical variation (incidentally, this is an argument against using M values, as described above). To see if our results were biased by variation in technical noise rather than biological noise, we looked at DNA methylation at sites we identified as being differentially variable across samples types (DVPs). Only 17,054 (8.8%) of identified DVPs were completely methylated (minimum beta value > 90%) or unmethylated (maximum beta value < 10%) in all sample types and therefore potentially affected by elevated technical variation. Furthermore, if we look at the percentage of probes that are completely methylated (minimum beta value > 90%) or unmethylated (maximum beta value < 10%) in all sample types, the percentage that are DVPs is much lower than the percentage across all probes (**Figure 2**). Given our careful experimental design, these results provide additional confidence that our results are minimally affected by technical variation.

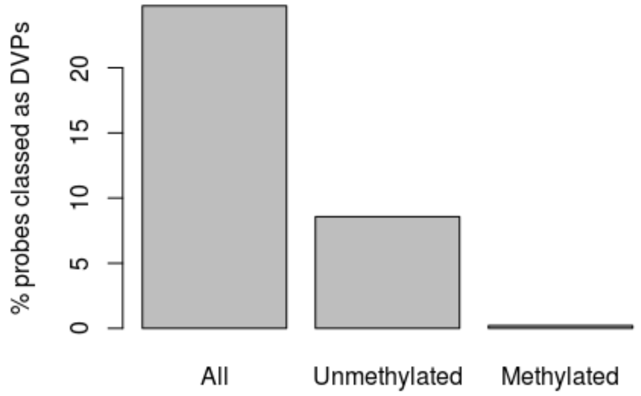

Figure 2. Bar chart showing the percentage of DNAm sites classed as differentially variable positions (DVPs) for i) all sites, ii) sites where the maximum DNA methylation level across all sample types is < 10% (i.e. unmethylated) and iii) sites where the minimum DNA methylation level across all sample types is > 90% (i.e. methylated).

iii) Concerns that we have not accounted for genetic variation in our analysis.

The goal of our analyses was not to identify the *source* of the variation we observe, but to characterise and describe how differences are reflected across sample types. The Reviewer

correctly summarises that genetic variation is an important factor known to influence variation in DNA methylation. In fact, we have previously shown that co-variation in DNAm between blood and brain is to some extent explained by shared genetic effects[8], which might indicate that shared genetic effects would lead to more similar levels of variability between cell types rather than different levels of variability. To explore the Reviewer's recommendation that we factor genetic variation into our analyses, we have taken advantage of a database of blood DNA methylation quantitative trait loci (mQTL) recently generated by our group in a large population cohort[9]. Comparing the DNAm sites identified as associated with mQTLs, and DNAm sites identified in this manuscript as being DVPs, we find that the percentage of DVPs that are mQTLs (18.6%) is virtually identical to the background percentage of all sites (18.8%). While this is an interesting analysis, it also raises the question of what other factors drive the differences in DNAm variability, for example environmental exposures. Therefore, we believe that adding this analysis to our manuscript will raise more questions than it answers but that this would be a good focus for future follow-up work and we have added a comment to this effect in the Discussion.

8) ***The Reviewer suggests our data has the potential to be applied to other EWAS findings in addition to smoking and BMI.***

We agree with the Reviewer that our data have broader utility for the interpretation of EWAS results beyond those from analyses of BMI and smoking. We have therefore performed additional analyses to assess EWAS data from other phenotypes. To facilitate this analysis we downloaded the complete database of results from the online EWAS catalog (http://ewascatalog.org/), which incorporates associations reported in the literature. Across studies undertaken in whole blood there were 104,945 significant DMPs ($P < 10^{-7}$) associated with 75 traits. 2,920 of these associations, involving 2,380 sites for 30 traits, involved DNAm sites that we identified as characteristic of a single blood cell type. We have added a Supplementary Table listing these results. To highlight enrichments, we calculated a fold change statistic for each trait and blood cell type to assess whether the number of characteristic sites was more (or less) than expected (**Figure 3**). This revealed some interesting patterns: sex, for example, had 431 associated sites that were characteristic of a single blood cell type with the distribution across blood cell types in line with the expected ratios. In contrast, DMPs associated with C-reactive protein were enriched for sites characteristic of granulocytes, and DMPs associated with chronic kidney disease were enriched for sites characteristic of monocytes. We have added these results to our manuscript.

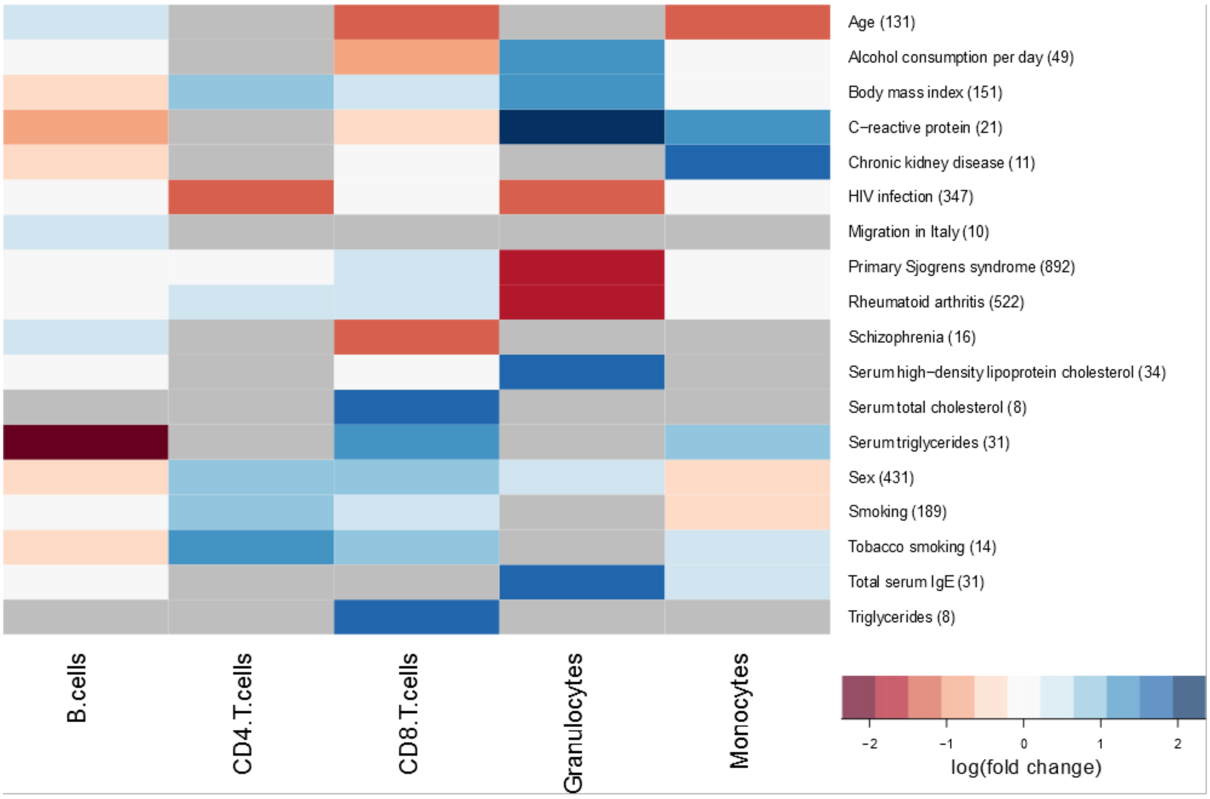

Figure 3 Heatmap showing the ratio of observed number to expected number of characteristic sites for each blood cell type across multiple EWAS traits. Association results were downloaded from the EWAS catalog (http://ewascatalog.org/) and DMPs filtered to those performed using whole blood and with a P < 1e-7. This heatmap contains all traits, where at least 5 significant associations were characteristic of a blood cell type. Grey squares indicate that there was no characteristic site for that cell type for that trait.

9) ***The Reviewer notes that the cumulative variance of specific cell types is the factor explaining whole blood variance and suggests that we include the measured percentage per cell type per individual.***

As requested in Comment 4, we have now added a summary of the number of cells sorted for each individual to our manuscript. We reiterate that the way in which our FACS isolation was performed means that it would be erroneous to interpret these as actual cell proportions and therefore it is inappropriate to include them as covariates in any analysis.

10) ***The Reviewer was confused about the "characteristic scores" we describe and wondered how applicable they might be to other studies. Instead they suggest that we use our data to generate a novel deconvolution algorithm.***

The Reviewer correctly identifies that our "characteristic score" methodology is not a substitute for deconvolution algorithms that estimate the proportions of blood cell types from a whole blood profile, but this was not the goal of our approach. Several excellent blood deconvolution methods for whole blood already exist. However, our data are freely available to be used as reference data for these deconvolution algorithms – in fact, they represent one of the first systematic analyses of blood cell-types using the Illumina EPIC array – and we have now highlighted this in the Introduction of our manuscript.

Our primary goal was to improve our understanding of how cell-specific profiles of DNAm influence measurements made in whole blood, with the aim of providing guidance about the interpretation of epigenetic analyses performed in whole blood. Typically, cell-specificity is defined as instances where a molecular mark is only present (or altered) in a single cell type, and absent in all other cell types. For example, in gene expression studies it would be

appropriate to statistically compare the mean level of expression across cell types to identify genes with cell-specific profiles. However, both the presence and absence of DNA methylation defines cell types, and the interpretation of these differences is dependent on genomic context. Furthermore, as the level of DNA methylation represents a measure across a heterogeneous population of cells, we do not think that the typical definition of cell-specificity is relevant for determining which cell type, trait-associated variation is attributable to. For example, in the scenario where DNA methylation is fully methylated in B-cells and unmethylated in all other blood cell types, the level of DNA methylation in whole blood will be proportional to the proportion of B cells in that sample. Furthermore, variation in whole blood across a sample will then correlate with the cellular composition of each sample, rather than the variance in DNA methylation within B cells.

Instead, we argue that it is more meaningful to identify instances where *variation* across a sample is specific to a single cell type, and therefore it can be assumed that the variation in whole blood is driven by variation within that cell type. We therefore designed a statistical framework to test whether the variance of DNAm in a single cell type is greater that the variance across all other cell types (after adjusting for the mean level of DNAm in that cell type). In order to define sets of CpGs that were specific to a single cell type, we used a stringent cut-off based on multiple testing correction for the whole array, and therefore we are confident that our results are robust. We acknowledge that our results may be incomplete; for example, our ability to make statistical comparisons of the magnitude of variation is limited by our sample size and the external sources of variation present in our sample, but we disagree with the notion that this analysis is not translatable to other studies.

11) ***The Reviewer suggests that our application of the characteristic scores to EWAS of BMI and smoking is the most interesting part of the manuscript, but asks if we can go further to distinguish between EWAS findings that are driven by a shift in cell type proportion and a cell-specific DNA methylation change.***

The Reviewer makes a very interesting suggestion, whereby a combination of cell-specificity (defined as differential DNA methylation levels in single cell type) and our characteristic scores (defined as elevated variation in a single cell type) can distinguish the two ways trait associated variation in bulk tissue can arise. The first scenario, where the association is purely driven by variation in cell composition that is correlated with the outcome would result in multiple CpGs specific for a single cell type being associated. The second scenario would lead to a more limited number of associations, potentially implicating multiple different cell types. Of course, it is not unrealistic that a combination of both scenarios may be at play for a single EWAS dataset.

We expanded the application of our characteristics scores to interpret existing EWAS to first consider additional traits (see response to Comment 8) but also to distinguish these two scenarios. First to identify instances of Scenario 1, we generated cell-specificity scores for all pairs of DNA methylation site and each cell type. These were calculated using a mixed effects regression model using data for the five purified blood cell types, using indicator variables to compare the level of DNAm in one cell type to the mean of the other four, controlling for individual and family ID as nested random effects, where a separate model was fitted for each cell type. Cell-specific scores were then generated for each DNAm site as the –log10(P-value)*sign(regression coefficient), so that significant increased DNAm level in a cell type would have a large positive score and significant decreased DNAm level in a cell type would have a large negative score. We compared the cell-specific scores to our characteristic scores, finding that while there were correlations within each type of score, there were negligible correlations between them, suggesting that they are picking up different patterns at different sets of DNAm sites (**Figure 4**). Therefore, we are confident that were we report specific CpGs associated with a trait to be specific to a single cell type, these are not false positives induced by heterogeneity of cellular composition. We have not added

this analysis to manuscript, but if the Reviewer thinks it provides a useful clarification we are happy to do so.

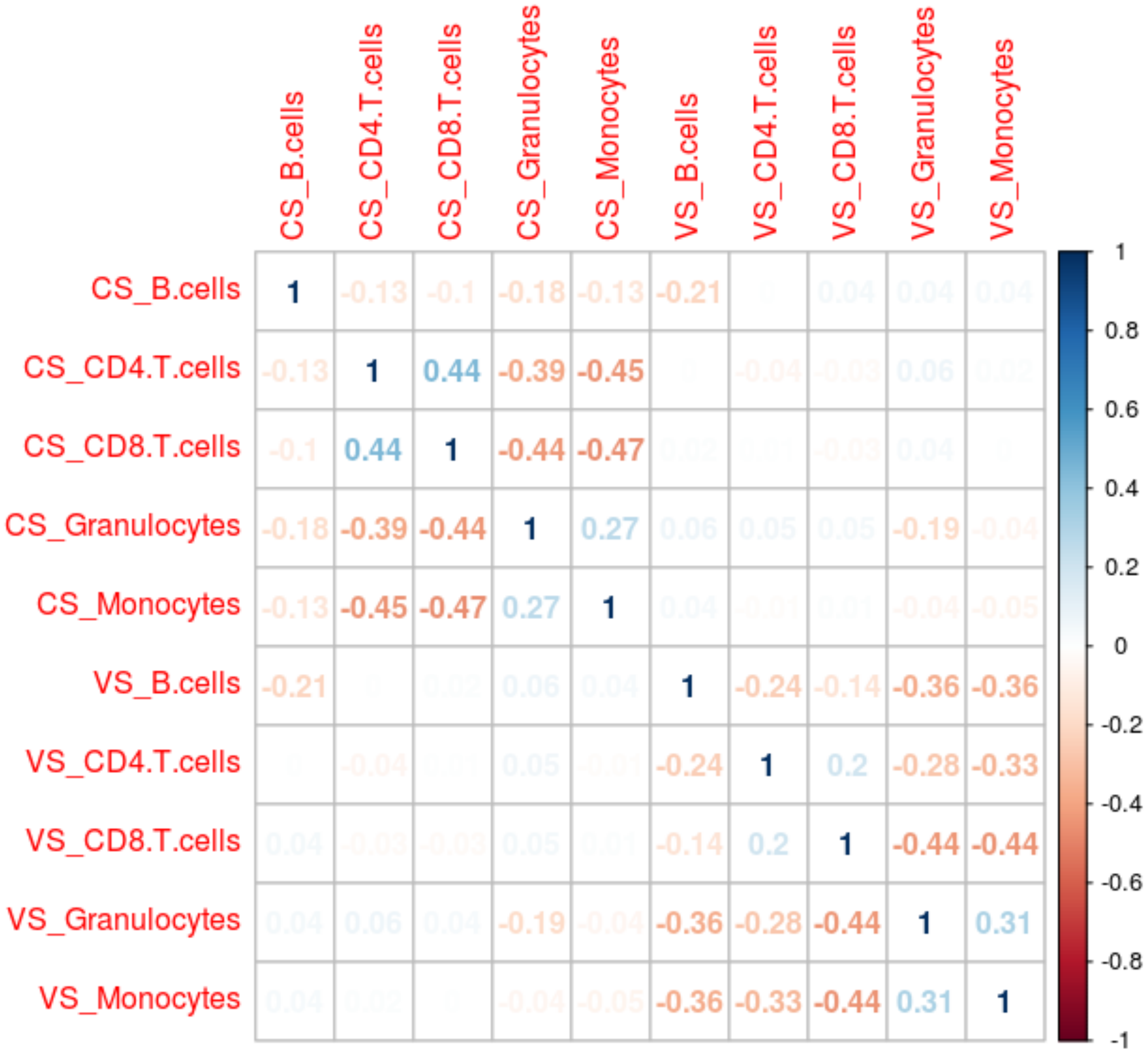

Figure 4. Correlation statistics between the blood cell type cell-specific (CS) and variance-specific (VS) scores.

Reviewer 2:

1. ***The Reviewer queries our statement that we have performed the largest study of purified blood cell types to date, citing a number of published studies which had larger samples of purified blood cell (Paul et al., 2016, Nat Comm; Ewing et al., 2019, EBioMedicine).***

The Reviewer is correct that our dataset is not necessarily the largest in terms of the number of individuals with DNAm data generated from purified blood cell types. However, our study performs the most comprehensive comparison of the major blood cell types by considering both differences in mean DNAm level and the variability of DNAm, and is the first to characterise how the profiles of the individual cell types combine to influence variation in whole blood. The studies the Reviewer refers to make few comparisons between cell types beyond comparing if disease-associated DMPs identified in their EWAS are shared between cell types. We would still argue that our study is novel and of broader interest to other researchers in the field of epigenetic epidemiology than the papers the Reviewer refers to. However, we agree that some of our statements were perhaps overstated and have therefore rewritten these sections to better contextualise our study.

2. ***The Reviewer notes that our finding that epigenetic clocks vary across tissues is not novel, citing a number of Review articles (e.g. Adam et al., 2019, Mol Cell; Bell et al., 2019 Genome Biology; Horvath and Raj, 2018, Nature Reviews Genetics) and raises a concern that the young age of our sample may be influencing our results.***

We agree that others have commented on the tissue-specific nature of epigenetic clocks and we have added some of the references the Reviewer mentions to our manuscript. The fact that the Reviewer only cites review articles that have discussed this issue, however, reinforces our original assertion that data comparing the performance across multiple tissues in the same individual, collected at the same time is lacking. The Reviewers' concern that the age of our samples (19 years old) may undermine our results is noted and we had already acknowledged this as a limitation in the Discussion.

3. ***The Reviewer highlights that there are other studies (Varley et al., 2013, Genome Research; Lowe et al., 2015 Epigenetics) which report blood DNAm profiles to be strikingly different from other tissues.***

We thank the Reviewer for bring these papers to our attention; indeed, these findings complement our results and we have now highlighted these previous analyses in our discussion.

Reviewer 3:

Major comments:

1. ***The Reviewer found our work to be of interest, but had a few concerns with our analysis, including:***

   i) The use of methods that did not control for the nested structure of our sample

This comment echoes one made by Reviewer 1 (Comment 3). As described above, we agree with the Reviewer that our original analysis was incomplete and we have therefore adjusted our analyses as far as possible to factor in the hierarchical nature of our samples, including replacing linear models with mixed effects models, where appropriate.

   ii) The experimental design and specifically how batches were designed, and whether we adjusted for age and sex.

As stated in response to Reviewer 1, we took great efforts to minimize batch effects in our data. A major batch effect when profiling DNAm using the Illumina EPIC array is ‘slide’, which incorporates 8 samples. Therefore, for the 8 sample types, all samples for a twin pair (n = 16) were run across two chips on the same plate, located adjacent to each other. For each twin pair the first slide contained the B-cells, CD4 T-cells, CD8 T-cells and monocyte samples and the second slide contained the whole blood, granulocytes, buccal and nasal samples, with samples of the same tissue/cell-type being run adjacent to each other. An example format is provided in the table below. As all the samples were the same age at the time of sampling, we did not control for age, neither did we adjust for sex. Given the matched nature of study design, we do not believe that sex will be a confounder in our analyses as it is balanced across the different sample types. Furthermore, having now included an individual and family random effect, if it was an issue it would be adjusted for in our revised analyses. Although sex effects are highly prevalent on the sex chromosomes (which we excluded prior to analysis), they are less of an issue on the autosomes and the magnitude of the effects between tissues and cell types are much larger than sex effects.

	Chip 1	Chip 2
R01C01	Bcells – Twin 1	Granulocytes – Twin 1
R02C01	Bcells – Twin 2	Granulocytes – Twin 2
R03C01	CD4 T-cells – Twin 1	Whole blood – Twin 1
R04C01	CD4 T-cells – Twin 2	Whole blood – Twin 2
R05C01	CD8 T-cells – Twin 1	Nasal – Twin 1
R06C01	CD8 T-cells – Twin 2	Nasal – Twin 2
R07C01	Monocytes – Twin 1	Buccal – Twin 1
R08C01	Monocytes – Twin 2	Buccal – Twin 2

   iii) Whether we excluded DNAm sites known to be affected by SNPs.

We did exclude DNAm sites known to be affected by common SNPs. This was done using the reference lists generated by McCartney et al[10] for the EPIC array. Specifically, we excluded any site which contains a SNP common (MAF < 5%) in the European population. While we did state this in the methods of the original manuscript, we apologize for not providing the reference of the paper from which we obtained the list; this has now been corrected in the revised submission.

2. ***The Reviewer questions our use of pairwise ANOVAs to identify DMPs and suggests we instead use a method that leverages information across the dataset. They also question our choice of multiple testing threshold for identifying significant DMPs.***

In light of the Reviewer's comment, we believe that they have misunderstood our analytical approach. We perform a single ANOVA for each site using all data available for that site, to get an overall p-value which we used to identify DMPs that have significantly different mean DNA methylation between tissues or cell types. The pairwise analysis they refer to, whereby we took whole blood as a baseline, was only relevant for the subset of sites identified by the ANOVA, in order to characterise the specific differences detected using ANOVA. They were not used to identify significant DMPs. As an ANOVA is omnibus test, it does not tell us anything about the significant difference it is capturing. Therefore, we needed a way to characterise the differences at the DMPs we detected, and hence used the t-tests we had already calculated. While we could apply post-hoc tests that perform all pairwise comparisons between all pairs of sample types, we felt this would complicate the presentation of the results. Instead taking whole blood as a reference category enabled us to draw conclusions to a common reference and allowed us to give an accurate summary of the differential DNAm patterns in our data.

We agree with the Reviewer that an appropriate multiple testing adjustment is an important consideration when performing an EWAS given the number of sites tested and the potential for false positives. We do not typically use an FDR approach as we have observed variable behaviour across studies due to its dependence on the distribution of p-values, which can vary between EWAS. We recently published a study[1] investigating what an appropriate threshold for EWAS conducted with the EPIC array would be and $P<9e-8$ was the threshold we identified as being optimal and therefore we believe that our significance threshold is justified and appropriate. As the Reviewer notes this is not that dissimilar to a Bonferroni multiple testing correction (and certainly more stringent than FDR) so we are confident that the proportion of false positives in our studies is adequately minimised.

3. ***The Reviewer was intrigued by our observation that "*****Overall, our results suggest that cell-specific levels of DNAm occur in a hierarchical manner, where at a subset of genomic loci profiles are shared between cells from the same linage"** ***and asked whether we could explore this further or find a better way to present this data for example using the upsetR package.***

We thank the Reviewer for drawing our attention to the *upsetR* package as an alternative way of presenting overlaps. We used it to generate a plot (**Figure 5**) which has been added to our manuscript as a Supplementary Figure. When we described our results as hierarchical, what we meant was that the majority of sites were shared, i.e. different in multiple cell types rather than specific to a single cell/tissue type, with the proportion shared being higher for more closely-related cell types/tissues. Indeed, the *upsetR* plot highlights that the most common intersect involves all samples types. The second most common intersect is sites that distinguish buccal and nasal from blood sample types. The third most common intersect distinguishes buccal, nasal and monocytes and granulocytes, while the fifth most common interest groups buccal and nasal with the B-cells and T-cells. So across these two groups of DMPs we can separate the three main groupings sample types. Using the additional intersects we can then continue to further segregation the sample types. Our conclusion is that signatures across a number of DNAm sites is needed to define cell types, and that the closer two cell/tissues are in terms of linage, an increasingly restricted set of sites is needed to distinguish them.

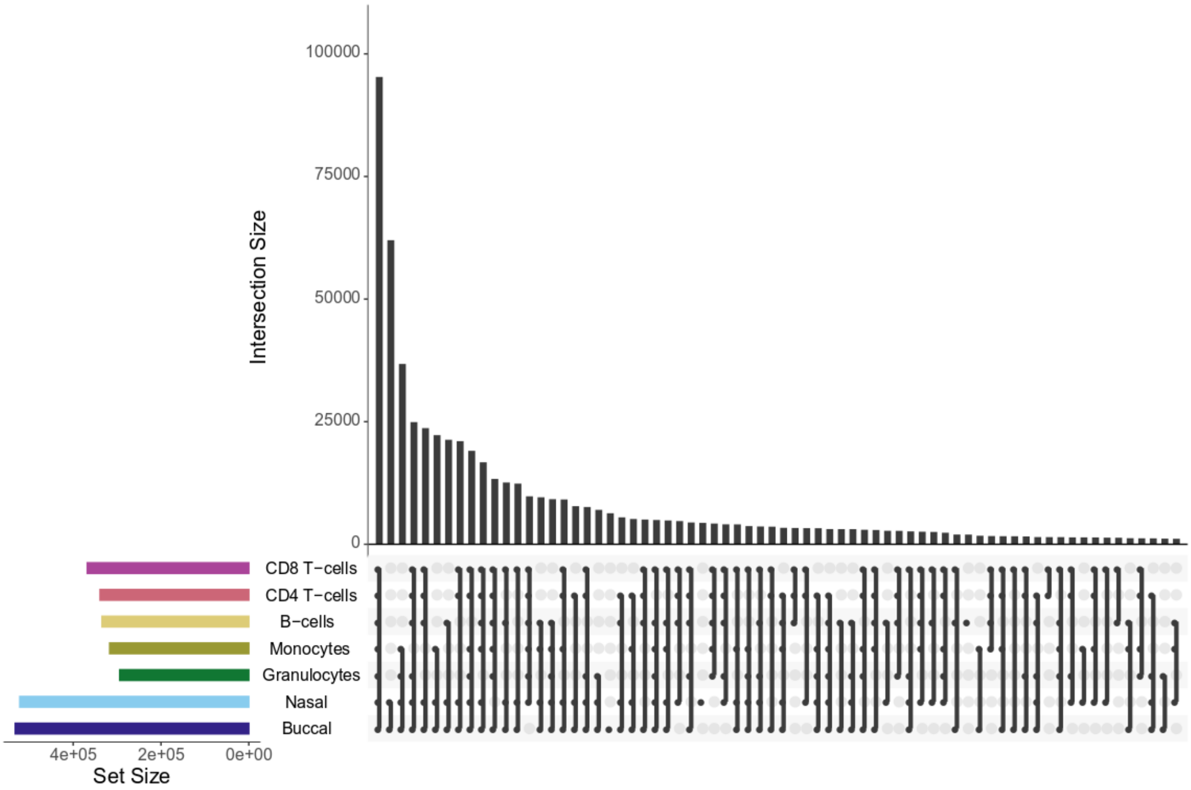

Figure 5. Histogram showing the most common intersects between cell- / tissue-types of sites differentially methylated between sample types. Taking all sites identified as having a significantly different level of DNA methylation in at least one cell or tissue type (n = 611,070; ANOVA $P < 9x10^{-8}$) we considered t-statistics comparing each sample type and whole blood to determine which cell/tissue types each site was significantly different in ($P < 0.05$). This plot identifies which combination of cell-/tissue types has the most shared DMPs, with the vertical histogram at the top indicating the number of shared DMPs and the matrix underneath highlighting which cell-/tissue types share those DMPs. The coloured bars in the horizontal histogram in the bottom left indicates the total number of DMPs for each cell-/tissue-type.

4. ***The Reviewer comments that it is not clear what comparisons we are describing when discussing the differences in ages predicted using the Horvath Epigenetic Clock.***

We thank the Reviewer for highlighting this confusion and we have edited the text to make it clear what the comparison is where mean difference statistics are presented.

5. The Reviewer asks for clarification on the number of DNAm sites included in the study and how those numbers were arrived at, given we state two different numbers in the Methods and Results.

We apologise for any confusion caused here. The number cited in the Methods (n = 802,216 DNAm sites) includes both the autosomes and sex chromosomes, whereas the number given in the Results (n = 784,726 DNAm sites) is just sites on the autosomes. For reference, the sex chromosomes were excluded after normalisation. We have updated our methods section to make this clearer.

Minor comments:

6. The Reviewer found our use of the word "optimal" in the sentence "principal component (PC) analysis was used to determine the optimal axes of variation" to be an unusual choice.

In this sentence we used the word 'optimal' to describe the top three PCs, which enabled all 8 sample types to cluster as distinct groups. We agree that the wording is perhaps confusing and have altered it to make the sentence clearer.

7. The Reviewer suggests that we are overly dismissive of the role that genetics may play in driving covariation between tissues. They think that we could be more nuanced in the analysis, but ultimately leave it to our discretion.

Our reference to genetic effects in the section on co-variation between cell-/tissue types was not intended to be dismissive of the role of genetic variation, but more aimed to explain the wider tail in the distribution of correlation coefficients. As we mention in our response to Reviewer 1 (Comment 7iii), genetic variation is likely to be one factor that underlies the co-variation in DNAm but we believe an extensive analysis of which sites are genetically mediated is beyond the scope of this manuscript.

8. The Reviewer suggests we implement a display friendly colour scheme.

This is a very welcome suggestion by the Reviewer, and we have implemented this change using the colour palette they suggested. Furthermore, we have also taken this opportunity to change the order of the sample types so that they now align with cell lineages. Specifically, they are now ordered as Buccal, Nasal, Whole Blood, Granulocytes, Monocytes, B-cells, CD4 T-cells and CD8 T-cells. We believe the figures now present our data in a much more accessible format.

9. Figure 1a - whole blood is not labelled in the legend.

We thank the Reviewer for highlighting this omission; whole blood has now been included in the updated figure legend.

10. The Reviewer suggests replotting Figure 1b as three 2d scatterplots.

As requested by the Reviewer we have changed Figure 1b to three two-dimension scatterplots each showing a pairwise combination of PCs.

11. The Reviewer highlights some vertical white lines in Supplementary Figure 7 and asked if they were compression artefacts or actual signal?

We thank the Reviewer for drawing our attention to this. They are indeed artefacts of the way the plots were combined into a single file. We have rectified this in the revision. We have also taken the opportunity to change the colour scheme for something more distinctive.

References

1. Mansell G, Gorrie-Stone TJ, Bao Y, Kumari M, Schalkwyk LS, Mill J, et al. Guidance for DNA methylation studies: statistical insights from the Illumina EPIC array. BMC Genomics. 2019;20(1):366. Epub 2019/05/14. doi: 10.1186/s12864-019-5761-7. PubMed PMID: 31088362; PubMed Central PMCID: PMCPMC6518823.

2. Ebrahimi P, Luthman H, McGuigan FE, Akesson KE. Epigenome-wide cross-tissue correlation of human bone and blood DNA methylation - can blood be used as a surrogate for bone? Epigenetics. 2020:1-14. Epub 2020/07/21. doi: 10.1080/15592294.2020.1788325. PubMed PMID: 32692944.

3. Herzog EM, Eggink AJ, Willemsen SP, Slieker RC, Felix JF, Stubbs AP, et al. The tissue-specific aspect of genome-wide DNA methylation in newborn and placental tissues: implications for epigenetic epidemiologic studies. J Dev Orig Health Dis. 2020:1-11. Epub 2020/04/24. doi: 10.1017/S2040174420000136. PubMed PMID: 32327008.

4. Varley KE, Gertz J, Bowling KM, Parker SL, Reddy TE, Pauli-Behn F, et al. Dynamic DNA methylation across diverse human cell lines and tissues. Genome Res. 2013;23(3):555-67. Epub 2013/01/16. doi: 10.1101/gr.147942.112. PubMed PMID: 23325432; PubMed Central PMCID: PMCPMC3589544.

5. Lowe R, Slodkowicz G, Goldman N, Rakyan VK. The human blood DNA methylome displays a highly distinctive profile compared with other somatic tissues. Epigenetics. 2015;10(4):274-81. doi: 10.1080/15592294.2014.1003744. PubMed PMID: 25634226; PubMed Central PMCID: PMCPMC4622544.

6. Slieker RC, Bos SD, Goeman JJ, Bovée JV, Talens RP, van der Breggen R, et al. Identification and systematic annotation of tissue-specific differentially methylated regions using the Illumina 450k array. Epigenetics Chromatin. 2013;6(1):26. doi: 10.1186/1756-8935-6-26. PubMed PMID: 23919675; PubMed Central PMCID: PMCPMC3750594.

7. Hearn NL, Coleman AS, Ho V, Chiu CL, Lind JM. Comparing DNA methylation profiles in saliva and intestinal mucosa. BMC Genomics. 2019;20(1):163. Epub 2019/02/28. doi: 10.1186/s12864-019-5553-0. PubMed PMID: 30819108; PubMed Central PMCID: PMCPMC6394071.

8. Hannon E, Lunnon K, Schalkwyk L, Mill J. Interindividual methylomic variation across blood, cortex, and cerebellum: implications for epigenetic studies of neurological and neuropsychiatric phenotypes. Epigenetics. 2015;10(11):1024-32. doi: 10.1080/15592294.2015.1100786. PubMed PMID: 26457534.

9. Hannon E, Gorrie-Stone TJ, Smart MC, Burrage J, Hughes A, Bao Y, et al. Leveraging DNA-Methylation Quantitative-Trait Loci to Characterize the Relationship between Methylomic Variation, Gene Expression, and Complex Traits. Am J Hum Genet. 2018;103(5):654-65. Epub 2018/10/25. doi: 10.1016/j.ajhg.2018.09.007. PubMed PMID: 30401456; PubMed Central PMCID: PMCPMC6217758.

10. McCartney DL, Walker RM, Morris SW, M. MA, J. PD, L. EK. Identification of polymorphic and off-target probe binding sites on the Illumina Infinium MethylationEPIC BeadChip. Genomics Data. 2016;9(September):22-4. Epub 26 May 2016. doi: http://dx.doi.org/10.1016/j.gdata.2016.05.012.
