# Supplementary material for: Assessing the co-variability of DNA methylation across peripheral cells and tissues: Implications for the interpretation of findings in epigenetic epidemiology
Source: PLoS Genet. 2021 Mar 19;17(3):e1009443. doi: 10.1371/journal.pgen.1009443 (PMC8011804; doi:10.1371/journal.pgen.1009443)
Supplement: Attachment — Submitted filename: RESPONSE TO REVIEWERS.pdf [file pgen.1009443.s034.pdf]

PGENETICS-D-20-00987R1 – response to reviewer comments

We are delighted that all three reviewers were impressed by our revised manuscript. We have addressed the remaining minor comments from Reviewer 1 and 3 as discussed below and hope you will now find our manuscript suitable for publication in *PLoS Genetics*.

Reviewer 1

I much appreciate the significant effort the authors put in the revision. They added various interesting analyses and included valuable remarks to guide the interpretation.

Thank you!

My main concern continues to be the biological relevance of the variance analysis and the role of heteroskedasticity. The point is that CpGs with a mean methylation (beta value) closer to 0.5 will be more variable not due to biological influences but a mathematical effect. For me the clearest example is that most meQTLs will, apart from showing statistically different methylation between genotypes, also show a difference in variance. The latter is not due to additional environmental influences, but just the mathematical phenomenon. I strongly disagree with the authors' assumption that a change in variance equals biology and the interpretation that it reflects differential effects of environment is speculative if not incorrect ("However, this reflects biology" / "We identify numerous differences in the variability of DNAm between tissue and cell types, indicating that differences in DNAm (induced by environmental exposures, for example) have variable effects across cell types and tissues."). In fact, Levene's test is sensitive to the mathematical source of heteroskedasticity. One way out would be to instead apply double generalized linear models (DGLM) which is specifically developed to address the issue.

We accept the Reviewer's point and are aware of potentially pervasive phantom variance QTLs for molecular traits with large-effect QTLs (as it is the case for DNA methylation data)[1, 2]; we agree that differences in variance observed between sample-types with different mean levels of DNAm may not only reflect biology. We apologize if this was worded a little strongly in our last rebuttal. We stand by our use of beta-values rather than M-values for the reasons given in our previous response but have included discussion of the potential limitation regarding the relationship between mean DNAm and variance.

We also thank the Reviewer for their suggestion that we apply double generalized linear models (DGLM), which model the mean and dispersion parameter jointly. For context, double generalized linear models (DGLM) allow for the estimation of the mean and variance parameters, which is performed iteratively, and P-values can be computed using a χ-square test, for example using the R package *dglm* (https://CRAN.R-project.org/package=dglm). These models are not straightforward to apply, however, and in fitting them we encountered algorithmic convergence problems of the iteratively reweighted least squares procedure, likely due to their dependence on *glm* results. This is a recognized shortcoming that makes DGLM cumbersome for genome-wide variance QTL analysis[3].

Additionally, a recent study by Staley et al[4] used simulations to show that approaches such as DGLM which assume normality of data result in extremely inflated type I error rates when applied to DNA methylation data (see **Figure S3 from Staley et al**, coped below). Importantly, these error rates can be significantly reduced after data normalization, but when using this transformation a mean effect can induce a variability effect and vice versa, as has been previously observed[1]. Because of these drawbacks such approaches are unlikely to be optimal for assessing variability nor joint mean and variability effects, especially as normalizing outcome levels to overcome this problem can induce effects that were not present prior to the transformation[1, 4], an issue we also thoroughly discuss in our Discussion section (lines 481-489).

To try to adequately address the reviewer's concern, we therefore applied the joint location-and-scale score test (JLSsc), developed by Staley et al[4]. This approach essentially combines a location test and scale test, while accounting for the correlation between these tests. The authors proposed to test the joint null hypothesis $H_0 = \beta = \delta = 0$ in the model specification:

$$y_i = \alpha + x_i'\beta + \varepsilon_i$$

$$(y_i - \bar{y})^2 = \lambda + x_i'\delta + u_i$$

where x_i is a (k_x) vector of exposures and $\bar{y}$ is the sample average of y_i. The full derivation of the parameters' estimators can be found in the Supplementary Text of the referenced article. Our results using this approach are

virtually identical to those we report using Levene's (99% of sites are in common) and we have therefore not included them in the revised manuscript.

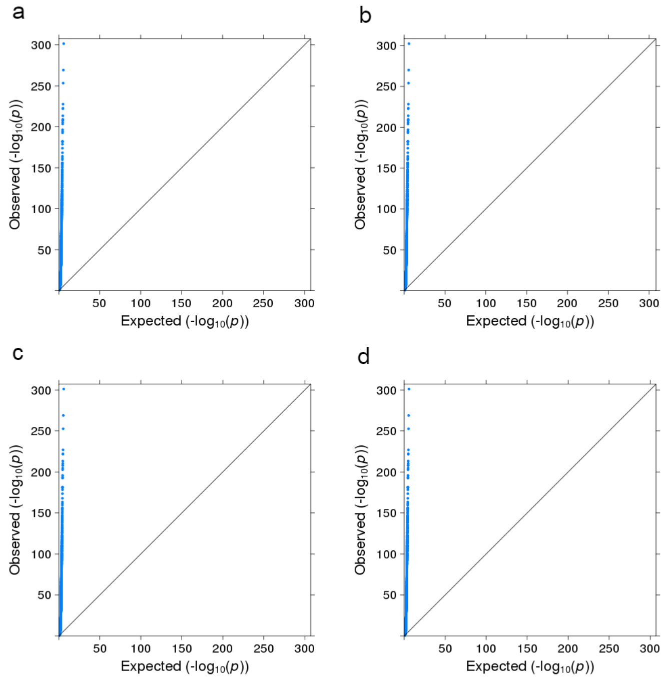

Figure S3 from Staley et al[4]: *QQ plots for type I error simulations for a binary exposure in 1000 samples. a, Bartlett's test (variability test). b, Likelihood ratio test (variability test). c, Likelihood ratio test (mean and variability joint test). d, DGLM (mean and variability joint test).*

The author state that: "The goal of our analyses was not to identify the source of the variation we observe, but to characterise and describe how differences are reflected across sample types." However, making the distinction between genetic and non-genetic sources seems crucial, in particular because the authors several times speculate about the role of environmental influences as an explanation for their findings. For example, genetic effects are likely to increase while environmental factors are likely to decrease correlation between tissues since the former is a pan-tissue phenomenon and an environmental; factor commonly affects a subset of tissues. If the authors are unable to measure a SNP array in the 30 individuals included in their analysis to directly correct for cis-methylation QTL effects, another indirect assessment is required.

The Reviewer makes an interesting comment, which prompted us to undertake some additional analyses to explore the extent to which covariation in DNAm between whole blood and constituent cell-types at individual sites is influenced by whether whole blood DNAm is influenced by genetic (or environmental) factors. We have previously characterized the proportion of variance in whole blood DNAm explained by additive genetic and environmental factors in a large cohort of monozygotic and dizygotic twin pairs, finding larger genetic influences at sites with variable and intermediate levels of DNAm[5]. We used this dataset to explore covariation in DNAm for the subset of sites where whole blood DNAm is under very strong genetic control (additive genetic effects on DNAm > 0.80, n = 6,617 sites), finding dramatically higher covariation in DNAm between whole blood and the individual blood cell-types compared to the levels seen across all sites in our dataset (see **Figure A** below). Across these sites, variation in DNAm in granulocytes explains a mean of 61.37% (SD = 34.67%) of the variance in DNAm in whole blood, with relatively higher levels also seen for the other individual blood cell types (mean variance explained by monocytes 57.09% (SD = 34.13%); B cells 50.75% (SD = 33.74%); CD4+ T cells 55.0% (SD = 34.87%); CD8+ T cells 51.91% (SD = 34.15%) (see **Figure B** below). We also found elevated levels of covariation in DNAm between whole blood and individual blood cell types for sites directly associated with a DNAm quantitative trait locus (mQTL) in whole blood using a previously published dataset generated by our group[6] (n = 147,683 sites). In contrast, we found that covariation was attenuated at sites where DNAm was strongly influenced by environmental factors (non-shared environmental effects on DNAm > 0.80, n = 143,501 sites) (see **Figure C** below). Across these sites, variation in DNAm in granulocytes explains a mean of only 4.31% (SD = 6.32%) of the variance in DNAm in whole blood, with equally low levels also seen for the other individual blood cell types (mean variance explained by monocytes 4.14% (SD = 5.99%); B cells 4.05% (SD = 5.72%); CD4+ T cells 4.41% (SD = 6.28%); CD8+ T cells 4.46% (SD = 6.23%) (see **Figure D** below). As the

Reviewer hypothesises, these results suggest that genetic effects act to increase correlation between cells/tissues while environmental factors act to decrease correlation, presumably because the former is a pan-tissue phenomenon and environmental factor commonly affect a subset of cells and tissues. We thank the Reviewer for suggesting these analyses and we have added the results to the revised manuscript.

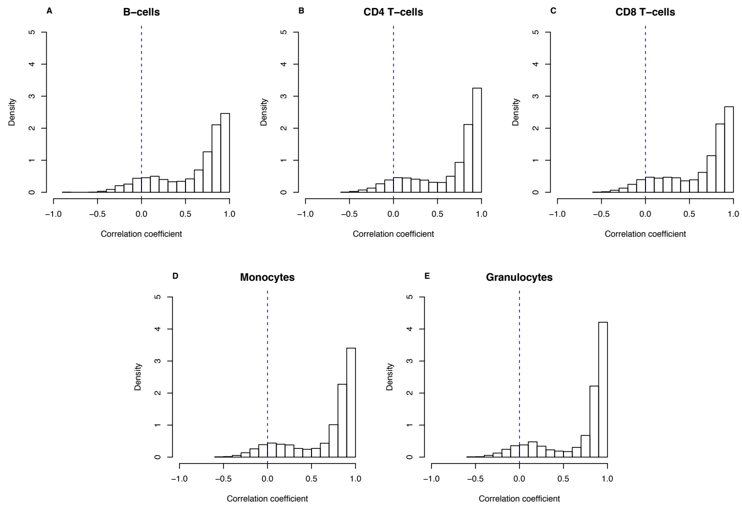

Figure A. Inter-individual variation in DNA methylation in whole blood is highly correlated with variation in isolated blood cell types for sites under strong genetic control. Histograms showing the distribution of correlation coefficients between DNA methylation in whole blood and the five blood cell types the subset of sites where whole blood DNAm is under strong genetic control (additive genetic effects on DNAm > 0.80, n = 6,617 sites) using estimates from Hannon et al[5]. **A)** B-cells, **B)** CD4 T-cells, **C)** CD8 T-cells, **D)** monocytes and **E)** granulocytes. The vertical blue dashed line indicates a correlation coefficient of zero. For all five cell types the distribution of correlation coefficients is dramatically skewed to the right.

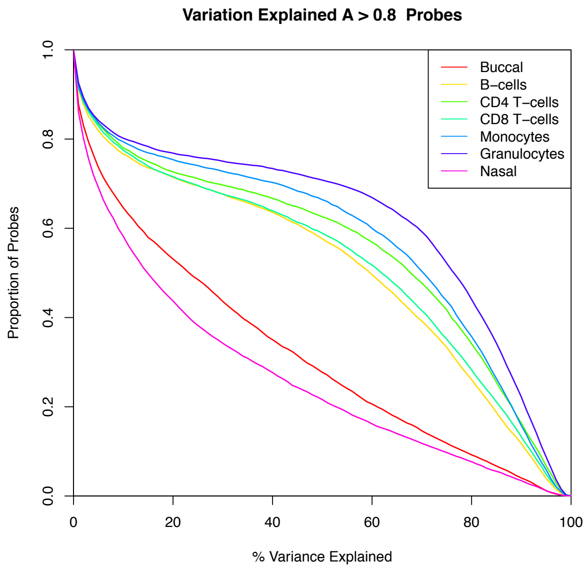

Figure B. Variation in DNA methylation in whole blood as a predictor of variation in the isolated blood cell types and other peripheral tissues across sites at which DNAm is under strong genetic control. Shown for the subset of sites where whole blood DNAm is under strong genetic control (additive genetic effects on DNAm > 0.80, n = 6,617 sites) using estimates from Hannon et al[5] is the proportion of sites (y-axis) for which variation in whole blood DNA methylation explains different levels of variance in five blood cell types (monocytes, granulocytes, $CD4^{+}$ T cells, $CD8^{+}$ T cells and B cells) isolated from the same individuals.

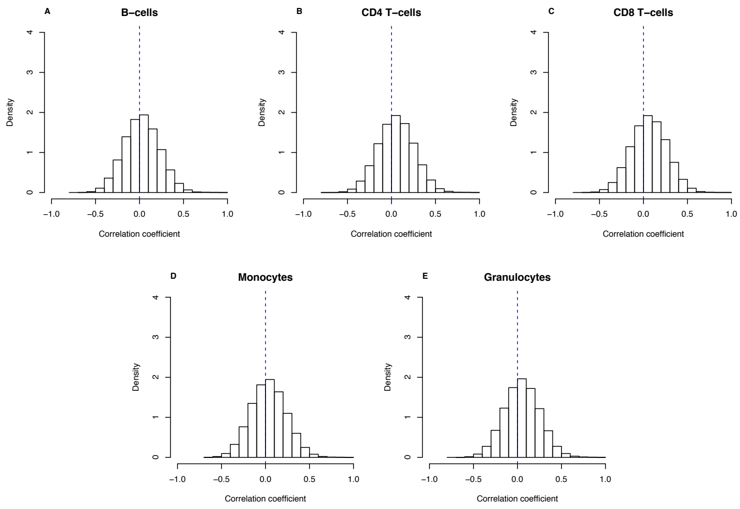

Figure C. Inter-individual variation in DNA methylation in whole blood is highly correlated with variation in isolated blood cell types for sites strongly influenced by non-shared environmental factors. Histograms showing the distribution of correlation coefficients between DNA methylation in whole blood and the five blood cell types the subset of sites where whole blood DNAm is strongly influenced by non-shared environmental factors (non-shared environmental effects on DNAm > 0.80, n = 143,501 sites) using estimates from Hannon et al[5]. **A)** B-cells, **B)** CD4 T-cells, **C)** CD8 T-cells, **D)** monocytes and **E)** granulocytes. The vertical blue dashed line indicates a correlation coefficient of zero.

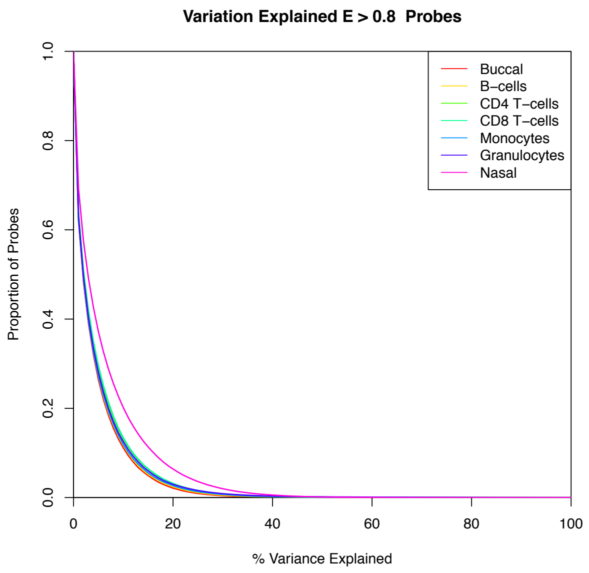

Figure D. Variation in DNA methylation in whole blood as a predictor of variation in the isolated blood cell types and other peripheral tissues for sites strongly influenced by non-shared environmental factors. Shown for the subset of sites where whole blood DNAm is strongly influenced by non-shared environmental factors (non-shared environmental effects on DNAm > 0.80, n = 143,501 sites) using estimates from Hannon et al[5] is the proportion of sites (y-axis) for which variation in whole blood DNA methylation explains different levels of variance in five blood cell types (monocytes, granulocytes, $CD4^+$ T cells, $CD8^+$ T cells and B cells) isolated from the same individuals.

In the Discussion the authors state 'therefore, isolating populations of cells is required to improve our understanding of the mechanisms underlying epigenetic dysregulation'. I think a more balanced conclusion is warranted on the basis of the data since the large majority of EWAS findings was not related to a cell-type specific effect according the analyses presented in the manuscript.

We have toned down this section of the discussion, but believe it remains important to stress benefits of purifying cell-types in epigenetic epidemiology.

May the fact that methylation in the major blood cell types explain only a proportion of whole blood methylation reflect the limitation mentioned in the discussion that many more blood cell (sub)types exist with varying abundance between individuals and DNA methylation levels? I guess that since whole blood methylation variation is not captured by main blood cell types, the main blood cell methylation variation does not fully capture variation in sub cell types.

Yes this is a good point, and we now further emphasize the importance of purifying additional cell types in the Discussion.

Reviewer 2

The manuscript is much improved and I have no major issues.

Thank you for the helpful comments in response to our original manuscript and we are delighted that you have no further concerns.

Reviewer 3

I thank the authors for their thorough response to my comments. I enjoyed rereading the manuscript, and the nuanced interpretation of the data presented within. As far as the figures go, the new colour scheme is much improved and easier to read, and the new 2D PCA plots look great and are also easier to interpret.

Thank you!

Batch effects and processing: I thank the authors for their clarification, but I am concerned by the strategy they reveal, which sets them up to confound technical and biological variation. From their answer to my original comment, samples from each twin pair were processed on different arrays, with samples from the same tissue from a single twin pair run on the same array. This means that if, eg, twin pair 5 is consistently different from all other twin pairs it is impossible to disambiguate whether the effect is driven by biology or technology. (For instance the couple of clustering failures and outliers in Fig1A in whole blood and nasal epithelium come in pairs - where they from the same ind/array?). I realise nothing can be done about it at this point, but I am curious to know if the authors included processing batches/array batches as covariates in their lme models. I realise that was more of a comment than a question.

As discussed in our previous responses, we actually feel we have controlled for technical variation as much as is possible in the design of the experiment. All tissue samples (whole blood, buccal and nasal samples) from both members of each twin-pair were collected at exactly the same time, and blood cell-sorting was performed immediately. All DNA samples were isolated in parallel, and sodium bisulfite treatment was performed at the same time in 96-well plates. Although samples were – by necessity given the numbers – spread over multiple Illumina chips, all chips were processed together in a single batch through all parts of the experiment on the same day using the same batch of reagents. As stated before, we included an ‘individual’ and ‘family’ random effect in our analysis models, which would further control for any residual confounding related to technical batch effects. Finally, given the magnitude of robust differences in DNAm between cell- and tissue-types, the results we present are likely to be resilient to any potential residual technical confounding.

Test statistics: The moderation of the multiple testing still seems insufficient to me. The authors perform ~700k lm/ANOVA (one per probe), then multiple t-tests (whole blood vs the seven other tissues) for those probes that ANOVA deems significant (~600k) to identify the relevant pairwise comparisons driving it, so 700k + (7*600k), which is a lot of tests. But the significance threshold for the t-test is set to 0.05 (inferred from one of the supp figure legends, since it is not reported in the methods) instead of 0.05/7 or similar. I note that in their response to me the authors claim that Tukey's HSD or similar would test too many comparisons (I don't necessarily disagree, it's a lot of pairwise comparisons!), but the current approach does not seem to me like a robust posthoc approach, and I would like to see more details on why the authors deem it appropriate to not even moderate the t-test p vals.

The Reviewer has slightly misinterpreted the way in which we have presented the results. In all cases we have used a stringent experiment-wide P-value (ANOVA $P < 9x10^{-8}$) to identify sites that are differentially methylated between sample-types. Across this subset of sites (all passing the stringent genome-wide correction), however, we wanted to then identify the very small sub-set of sites that showed evidence of differential DNA methylation in only a single sample-type compared to whole blood. Here we are *not* identifying additional differentially

methylated sites but distinguishing between differences that are either 'unique' or 'common' across sample-types. A lower P-value threshold here would in fact increase our chances of classifying a site as being 'unique', and we would therefore argue that our approach is in fact more stringent in identifying unique sites. Finally, given the number of cell types tested, it is easier to be significant in the ANOVA than the t-test due to different numbers of degrees of freedom. We have now rephrased the text and legend slightly so this is clearer.

line 175: The authors should state here that their samples come from twin pairs. This colours the reading of all subsequent results. This should also be made clearer in line 649.

Done

line 582: alluded, not eluded, I hope!

Done

line 774: publically should be publicly

Done

line 774: I looked up the GEO accession and find it only leads to the data from the purified blood cells, but not to the whole blood, nasal or buccal cells?

We apologize for this and have updated the GEO accession so it is now complete.

Figure 1: I couldn't help but notice that the clustering is ever-so-slightly different between the version of the figure in the original submission and in this current version. Anything interesting happening there?

The Reviewer has sharp eyes! When we went to replot the figures using the new colour scheme as requested by the Reviewer the clustering analysis kept crashing when using data for 5,000 probes. We therefore replotted the figure for the top 1,000 probes instead, as indicated in the legend.

Figure 2: Totally optional but a dashed line at 19 indicating the true sample age might be a nice touch.

This is a good suggestion and has been done.

References

1. Ek WE, Rask-Andersen M, Karlsson T, Enroth S, Gyllensten U, Johansson A. Genetic variants influencing phenotypic variance heterogeneity. Hum Mol Genet. 2018;27(5):799-810. Epub 2018/01/13. doi: 10.1093/hmg/ddx441. PubMed PMID: 29325024.

2. Wang H, Zhang F, Zeng J, Wu Y, Kemper KE, Xue A, et al. Genotype-by-environment interactions inferred from genetic effects on phenotypic variability in the UK Biobank. Sci Adv. 2019;5(8):eaaw3538. Epub 2019/08/28. doi: 10.1126/sciadv.aaw3538. PubMed PMID: 31453325; PubMed Central PMCID: PMCPMC6693916.

3. Dumitrascu B, Darnell G, Ayroles J, Engelhardt BE. Statistical tests for detecting variance effects in quantitative trait studies. Bioinformatics. 2019;35(2):200-10. Epub 2018/07/10. doi: 10.1093/bioinformatics/bty565. PubMed PMID: 29982387; PubMed Central PMCID: PMCPMC6330007.

4. Staley JR, Windmeijer F, Suderman M, Lyon MS, Smith GD, Tilling K. A robust mean and variance test with application to high-dimensional phenotypes. bioRxiv. 2020:2020.02.06.926584. doi: 10.1101/2020.02.06.926584.

5. Hannon E, Knox O, Sugden K, Burrage J, Wong CCY, Belsky DW, et al. Characterizing genetic and environmental influences on variable DNA methylation using monozygotic and dizygotic twins. PLoS genetics. 2018;14(8):e1007544. Epub 2018/08/10. doi: 10.1371/journal.pgen.1007544. PubMed PMID: 30091980; PubMed Central PMCID: PMCPMC6084815.

6. Hannon E, Gorrie-Stone TJ, Smart MC, Burrage J, Hughes A, Bao Y, et al. Leveraging DNA-Methylation Quantitative-Trait Loci to Characterize the Relationship between Methylomic Variation,

Gene Expression, and Complex Traits. Am J Hum Genet. 2018;103(5):654-65. Epub 2018/11/08. doi: 10.1016/j.ajhg.2018.09.007. PubMed PMID: 30401456; PubMed Central PMCID: PMCPMC6217758.
